# Quinine and Quinidine Derivatives as Photosensitizers for Photodynamic Inactivation of Bacterial Pathogens

**DOI:** 10.1021/acs.jnatprod.5c00570

**Published:** 2025-08-12

**Authors:** Irena Maliszewska, Anna Zdubek, Błażej Dziuk, Przemysław Boratyński

**Affiliations:** a Department of Organic and Medicinal Chemistry, Faculty of Chemistry, 214839 Wrocław University of Science and Technology, Wybrzeże Wyspiańskiego 27, 50-370 Wrocław, Poland; b Institute of Advanced Materials, Faculty of Chemistry, Wrocław University of Science and Technology, Wybrzeże Wyspiańskiego 27, 50-370 Wrocław, Poland

## Abstract

This study reports the synthesis and characterization of 5-azoniabenzo­[*no*]­tetraphene derivatives of *Cinchona* alkaloids with potential applications in antimicrobial photodynamic therapy (aPDT). These compounds exhibit absorption maxima at 430–441 nm, as well as in the UV-A (340–342 nm) and UV–C (270–275 nm) regions, with fluorescence emission peaks ranging from 519 to 534 nm. At nontoxic concentrations of 2 μg mL^–1^ and upon irradiation with blue light (418 nm), these compounds demonstrated potent bactericidal activity depending on the light dose. *Staphylococcus aureus* was eradicated after 5 min of irradiation (50 J cm^–2^) with the isomer of 7*R*,2’*S* configuration (**QN-1**) as a photosensitizer. Gram-negative bacteria exhibited reduced sensitivity, with *Acinetobacter baumannii* requiring an energy dose of 150 J cm^–2^ for effective killing, whereas *Proteus mirabilis* showed no significant reduction after exposure to 200 J cm^–2^. The differences in sensitivity are attributed to the structure of the cell envelopes, which influence the uptake of the photosensitizer: the compounds accumulated intracellularly in Gram-positive bacteria but remained extracellular in Gram-negative rods. It is suggested that the aPDT mechanism involves a combination of oxidative pathways for **QN-1** and **QD-1** as photosensitizers and a Type II mechanism when **QN-2** was applied.

## Graphical abstract

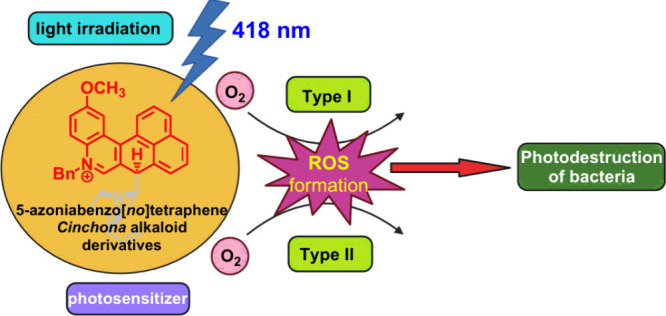

Quinine and its naturally occurring stereoisomer, quinidine, are natural alkaloids found in the bark of the *Cinchona* tree, which is native to South America.^1^ These compounds exhibit a broad spectrum of pharmacological activities. Their most well-known property is antimalarial activity, which has remained evident nearly for 400 years after Jesuit priests first documented its efficacy.^2,3^ These alkaloids have a similar mechanism of action as antimalarials (with quinidine reportedly being more effective in this regard), interfering with the ability of *Plasmodium* to grow and reproduce in human red blood cells by inhibiting the parasite’s ability to degrade hemoglobin to obtain essential amino acids for building its own proteins and for energy metabolism.^4^

In addition to their antimalarial activity, quinine and quinidine stabilize cardiac rhythm; however, only quinidine is used to treat this dysfunction.^5^ A primary mechanism of quinidine’s activity involves its interaction with cardiac ion channels. It predominantly inhibits sodium and potassium channels, thereby helping to stabilize the cardiac rhythm.

Ramić et al.^6^ synthesized a series of 46 *Cinchona* alkaloid derivatives differing in the position of a fluorine atoms within the molecule. It was demonstrated that all tested compounds reversibly inhibited human acetylcholinesterase (AChE) and butyrylcholinesterase (BChE) in the nano- to micromolar range.

It was also demonstrated that quinine derivatives exhibit antibacterial activity and can inhibit bacterial topoisomerase II (DNA gyrase).^7^ Another derivative of a *Cinchona* alkaloid, optochine, has shown highly selective antibacterial activity against *Streptococcus pneumoniae*. Several optochine derivatives were synthesized, and resistance studies combined with molecular modeling suggested that these compounds likely interact with the C ring of ATP synthase near the conserved glutamate binding site.^8^

Furthermore, quinine dimers have been shown to reversibly inhibit P-glycoprotein-mediated resistance to paclitaxel, thereby exerting an antiproliferative effect on cancer cells.^9^

Research has also shown that quinine and its derivatives have many unique biological effects for use in agriculture. To date, insecticidal activity^10–12^ and antifungal activity,^13^ including the inhibition of significant phytopathogens,^14,15^ have been documented.

Quinine is also found in certain foods and beverages, including tea, bitter lemon, and tonic water.

In this study, *Cinchona* alkaloid derivatives were assessed as potential natural photosensitizers (PS)—crucial components in the antimicrobial photodynamic inactivation (aPDI) of pathogens. This technique employs light-activated compounds (PS) that in the presence of molecular oxygen generate highly cytotoxic reactive oxygen species (ROS). The selection of an appropriate photosensitizer is essential as it markedly influences both the effectiveness of pathogen eradication and the likelihood of adverse side effects associated with the treatment. Antimicrobial photodynamic inactivation is a well-known nonantibiotic approach for the eradication of various pathogenic microorganisms (bacteria, viruses, fungi, and even protozoa).^16^ With increasing antibiotic resistance, aPDI presents a compelling alternative or complement to traditional antibiotic therapies. Notably, this technique effectively targets antibiotic-resistant pathogens,^17^ and bacteria are significantly less likely to develop resistance to aPDI than to classical antibiotic therapies.^18^ Furthermore, the combination of photodynamic antimicrobial inactivation with traditional antibiotics may help overcome resistance mechanisms and improve therapeutic outcomes.^19^

The photobactericidal properties of quinine were first reported over a century ago by Tappeiner, who studied its effects on *Bacillus pyocyaneus*, as well as on the protozoa *Paramecium caudatum* and *Amoeba proteus* when exposed to ultraviolet (UV) light.^20^ In particular, UVA irradiation (14 J cm^–2^) sensitizes quinine, resulting in complete photokilling of *Candida albicans* after a 24-h exposure period.^21^ Furthermore, research conducted by Morten et al.^22^ revealed that quinine, with absorption maxima at 331 and 360 nm, generates singlet oxygen upon UV irradiation, achieving a maximum photochemical efficiency of 36% in D_2_O at pH 7. This photoactive compound has been shown to oxidize amino acids such as histidine and tryptophan, leading to observable photo-cross-linking of calf lens proteins.

Despite the significant role of quinoline alkaloids in medicine and the availability of various synthetic routes for the development of quinoline-based compounds, their potential as photosensitizers remains underappreciated, primarily due to their absorption characteristics in the ultraviolet range.

As previously reported, a molecular rearrangement allows for the successful annulation of naphthalene and quinoline rings within *Cinchona* alkaloids. This transformation resulted in the formation of the novel 7*H*-5-azabenzo­[*no*]­tetraphene ring system, characterized by the presence of a sp^3^-hybridized carbon atom.^23^ The structural components of both the heterocyclic and heteroaromatic segments exhibit similarities to known biologically relevant compounds, suggesting potential interactions with proteins.^24–26^

In this study, five new 5-azoniabenzo­[*no*]­tetraphenes differing in the type of quaternary ammonium substituent, quaternization of the peripheral quinuclidine, and absolute configuration at two positions were synthesized. A comprehensive photochemical and photophysical study of these molecules was conducted, and their light-induced toxicity against Gram-positive and Gram-negative pathogenic bacteria such as *S. aureus*, *P. mirabilis*, and *A. baumannii* was evaluated.

## Results and Discussion

### Synthesis and Photochemical and Photophysical Studies

A unique 7*H*-5-azabenzo­[*no*]­tetraphene ring system has recently emerged from our laboratory.^23^ The only known examples of this system are compounds derived from *Cinchona* alkaloids in an unusual radical domino cyclization reaction. They incorporate the quinuclidine bicyclic ring of the original alkaloids and retain their stereochemistry. Preliminary experiments with these compounds have shown nonuniform fluorescence distribution in plant cells. Their emission wavelength differed between the electrically neutral 5-aza and cationic 5-azonia species, resulting in blue emission in neutral solutions and yellow-green emission in acidic ones. Solubility in nonacidified water remained poor. In order to modify both the solubility and fluorescent properties, basic azabenzo­[*no*]­tetraphenes derived from *Cinchona* alkaloids quinine (**QN-0**) and quinidine (**QD-0**) were alkylated separately with benzyl bromide and methyl iodide in fair yields (Figure 1A). The reactions with excess alkyl halides resulted in double nitrogen quaternization. In contrast to unmodified *Cinchona* alkaloids, the alkylation occurs preferentially at the aromatic nitrogen atom. This rather unusual reactivity was observed by the aligning patterns of ^1^H NMR signals in the aromatic part for **QN-1** and **QN-2** and the aliphatic parts for **QN-0** and **QN-1** (Figures S5–S9, Supporting Information). Relevant HMBC correlations of the benzyl protons with the quinoline part further supported the structural assignment. Finally, the X-ray structure of **QN-1** unambiguously confirmed both the tentative structure of the starting material and the alkylation site (Figure 1B) (see: Supporting data for the X-ray structure of **QN-1**; SI). In the crystal lattice, the conjugated aromatic systems (naphthalene and quinolinium) were oriented at ca. 25° angle, assuming *P* configuration within the helical ring system. This ensures a fair degree of conjugation between these aromatic systems. Overall, five new 5-azoniabenzo­[*no*]­tetraphenes were synthesized, differing in the type of quaternary ammonium substituent, quaternization of the peripheral quinuclidine, and absolute configuration at two positions (8 and 9 of the former alkaloid part) (Figure 1).

**Figure 1:**
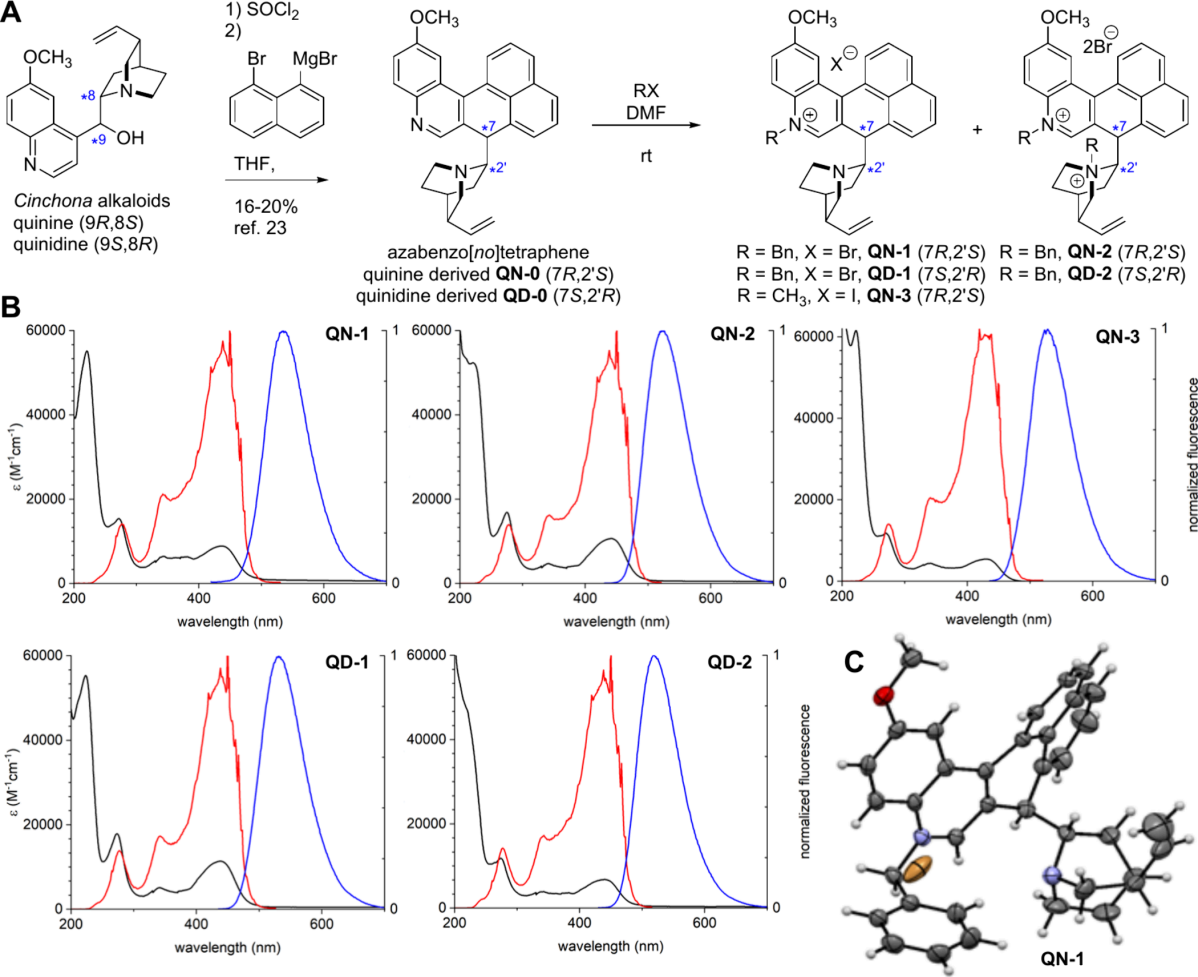
(A) Synthesis of *Cinchona* alkaloid-derived 5-azoniabenzo­[*no*]­tetraphenes; (B) UV–vis (black line), fluorescence excitation (red line) and emission (blue line) spectra for 10^–5^ M solutions of products in 1% aqueous ethanol; (C) X-ray structure of **QN-1**. One molecule is shown; thermal ellipsoids are set at 50% probability.

Compounds **QN-1** and **QD-1** remain sparingly water-soluble, while compounds **QN-2** and **QD-2** displayed rather good solubility. For compound **QN-2**, the solubility was measured spectrophotometrically at 2.5 mg mL^–1^ (4.2 × 10^–3^ M). All of the compounds display overlapping absorption in the visible range with maxima at 430–441 nm (log *ε* 3.7–4.1), in the UV-A range at 340–342 nm (log *ε* 3.6–3.8), and in the UV–C range at 270–275 nm bands (log *ε* 4.1–4.3). The studied quaternary salts display fluorescent properties similar to those of the protonated parent compounds (**QN-0** and **QD-0**). The excitation spectra were nearly identical for all compounds except for the methylated compound **QN-3**. The maximum emission wavelength varied between 519 and 534 nm (Figure 1, panel B, Table 1). Their fluorescence quantum yields were in the 0.12–0.34 range.

**Table 1:** Fluorescence Properties of Tested Compounds and Estimated Quantum Yields

compound	λ_max_ (excitation) nm	λ_max_ (emission) nm	φ_fl_
**QN-1**	434	534	0.195
**QN-2**	438	523	0.348
**QN-3**	428	527	0.143
**QD-1**	433	531	0.125
**QD-2**	433	519	0.165

Our screening studies (Tables S1–S3; SI) indicated that compounds **QN-1**, **QN-2**, and **QD-1** used as photosensitizers showed a high bacteriotoxicity, and thus only these salts were analyzed in subsequent experiments.

The kinetic profiles of photobleaching of the synthesized quaternary salts were next analyzed. Photostability is considered a key advantage of photosensitizers used in the light-induced destruction of pathogenic cells, and the photoinduced singlet oxygen generation cycle has always been considered to correlate with photosensitizer efficiency. The results obtained (Figure 2) show variations in the absorption spectra of **QN-1**, **QN-2**, and **QD-1** under irradiation with blue light, measured at intervals of 2–5 min. The decrease in the absorption bands of compounds **QN-1** and **QD-1** with increasing exposure time is not significant, and after 20 min of light exposure, it amounted to approximately 9% and 7%, respectively. No changes in the position of the absorption bands of these compounds were observed either. Only in the case of **QN-2**, the characteristic absorption band at a wavelength of 440 nm gradually decreased, broadened, and the maximum shifted toward a longer wavelength with increasing irradiation time (*k* = 0.027 s^–1^). After 20 min of irradiation, the absorption maximum was visible at a wavelength of 467–470 nm. The decrease in the absorption band of compound **QD-2** during the 20 min of exposure was approximately 42%. Therefore, in the next part of our experiments, a classical qualitative assay for reactive oxygen species based on the quenching of the fluorescence of tryptophan residues in albumin was used to study the effect of this salt. At a 290 nm excitation wavelength, independent fluorescence from both albumin in the 300–400 nm region and **QN-2** in the 450–700 nm range was observed (Figures S1–S2; SI). Irradiation from a 57.7 mW blue light source (418 nm) resulted in a rapid loss of fluorescence from albumin (*k* = 0.0072 s^–1^) and thus is indicative of the formation of reactive species. On the other hand, the loss of fluorescence attributed to the **QN-2** fluorophore occurred at a much slower rate (*k* = 0.0023 s^–1^) suggesting rather good resistance to photobleaching (Figure S1; SI).

**Figure 2:**
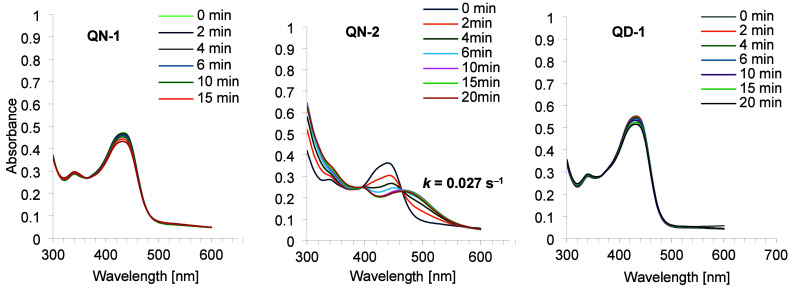
Monitoring visible light irradiation (418 nm) of **QN-1**, **QN-2**, and **QD-1** for 20 min with absorption spectra.

The two photochemical pathways known as Type I and Type II processes that the excited photosensitizer can undergo are then analyzed. The increase in absorbance values of the formed formazan over the time of exposure to light confirmed the potential for generating superoxide anion by electron transfer from the triplet state photosensitizers (**QN-1**, **QN-2**, and **QD-1**) to molecular oxygen, thereby indicating the feasibility of Type I mechanisms (Figure 3, panel A). The generation of singlet oxygen (Type II mechanism) was studied by using several analytical techniques. The intense time-dependent photooxidation of tryptophan in the presence of **QN-1**, **QN-2**, and **QD-1** indicated the potential for singlet oxygen generation (Figure 3, panel A). To verify the generation of singlet oxygen by the studied salts, the chemical probe 9,10-anthracenediyl-bis­(methylene)­dimalonic acid (ABDA) was also used. Chemical measurements using ABDA showed a significant decrease in absorbance at 402 nm for **QN-1**, **QN-2**, and **QD-1** after blue light irradiation (418 nm), suggesting a high efficiency of reactive ^1^O_2_ generation (Figure 3, panel A). The results obtained indicated that, up to 10 min of irradiation, there were no significant differences in the efficiency of singlet oxygen production among all the salts studied. However, with extended exposure to light (up to 25 min), the salt **QN-1** demonstrated the highest potential for singlet oxygen generation.

**Figure 3:**
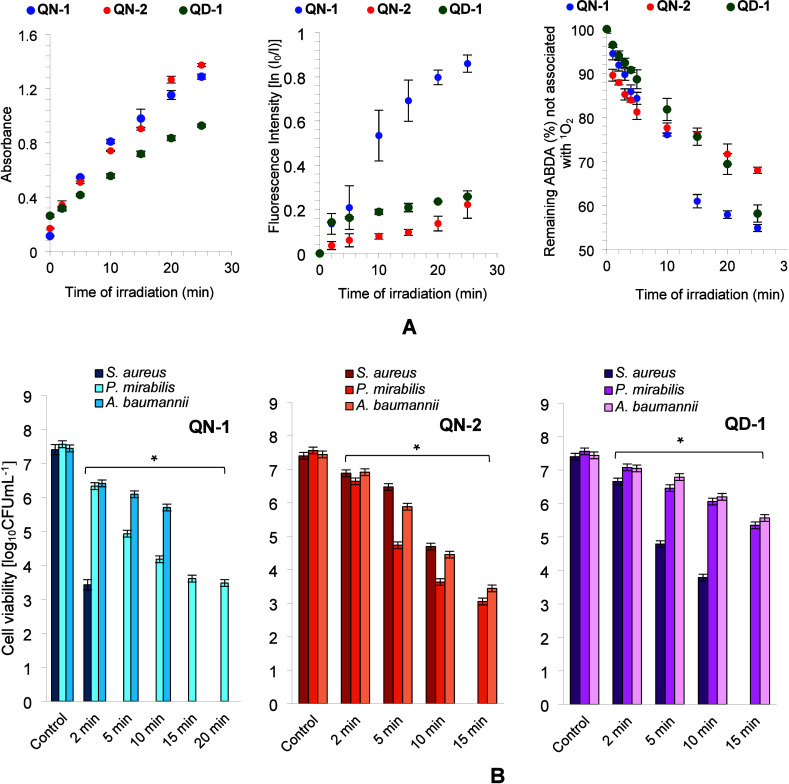
(A) Changes in formazan absorbance at 560 nm after different irradiation times in the presence of **QN-1**, **QN-2**, and **QD-1** (left chart); changes in fluorescence intensity of tryptophan after different irradiation times in the presence of **QN-1**, **QN-2**, and **QD-1**; photobleaching of ABDA probe after interactions with singlet oxygen generated by **QN-1**, **QN-2**, and **QD-1** (after light excitation) (right chart); (B) effect of **QN-1** 1997B, **QN-2** 1997C, and **QD-1** 1999B on the viability of studied bacteria after 2; 5; 10; 15; and 20 min of irradiation with blue light (418 nm).

The analysis of singlet oxygen generation efficiency following exposure of the tested salts **QN**-**1**, **QN-2**, and **QD-1** to blue light was also conducted by using the singlet oxygen sensor green (SOSG) probe. SOSG is a commercially available fluorescent probe characterized by high specificity for singlet oxygen (^1^O_2_), with negligible affinity for other reactive oxygen species (ROS). This probe consists of an anthracene moiety serving as an electron donor, which quenches the fluorescence of the attached fluorochrome (electron acceptor) via an electron transfer mechanism. Upon trapping of ^1^O_2_, the anthracene moiety forms an endoperoxide, rendering the oxygenated part incapable of functioning as an intramolecular electron donor, thereby restoring the fluorescence signal. SOSG exhibits weak blue fluorescence under basal conditions; however, upon reaction with ^1^O_2_, it emits a prominent green fluorescence at 525 nm. As shown in Figure S3 (SI), an increase in the SOSG fluorescence intensity was observed over the irradiation time, confirming the generation of singlet oxygen by **QN-1**, **QN-2**, and **QD-1** after exposure to blue light. The intensity of SOSG fluorescence depended on the type of salt tested and the irradiation time, with the highest value observed for **QN-1**. In contrast, the lowest SOSG fluorescence intensity was recorded for **QN-2**, indicating its lowest singlet oxygen generation capacity compared to **QN-1** and **QD-1**.

The effect of reactive oxygen species (ROS) quenchers, specifically azide ions and d-mannitol, was investigated *in vitro*, utilizing a range of tested salts as photosensitizers (Tables S4 and S5; SI). *S. aureus* cells treated with **QN-1** and **QD-1**, followed by exposure to blue light, exhibited a reduction in cell viability of approximately 3.97 and 3.61 log units (±0.02), respectively. In contrast, cells subjected to the same light irradiation conditions in the presence of azide ions demonstrated a decrease in the mortality rate by 3.26 and 3.53 log units (±0.02), respectively. Notably, this protective effect of azide ions was not observed when **QN-2** was employed as the photosensitizer. A comparable inhibitory effect on the phototoxic activity of **QN-1** and **QD-1** was also documented for *A. baumannii*. The reduction in viable cell counts of this microorganism after blue light irradiation with **QN-1** or **QD-1** was quantified at 1.35 ± 0.02 and 1.49 ± 0.2 log units, respectively. Under identical experimental conditions, the presence of azide ions led to a decrease in the mortality rate to 1.15 ± 0.02 and 1.36 ± 0.02 log units. This notable effect was again absent when **QN-2** was used as the photosensitizer.

The eradication of the studied pathogens in the presence of d-mannitol using **QN-1**, **QN-2**, and **QD-1** as photosensitizers also demonstrated partial inhibition of the photokilling effect (Table S5; SI). Furthermore, no cytotoxic effects were detected in cells treated with reactive oxygen species (ROS) quenchers, whether administered alone or in conjunction with the photosensitizer, when maintained in the dark (data not shown).

Generally, analysis of the produced cytotoxic ROS species by **QN-1**, **QN-2**, and **QD-1** in the presence of blue light showed that toxic superoxide anions as well as singlet oxygen may be generated during photokilling, suggesting that a mixed mechanism underlying the destruction of pathogenic cells is possible. Partial inhibition of the photokilling effect of **QN-1** and **QD-1** in the presence of quenchers (azide ions and d-mannitol) supports these assumptions. In experiments utilizing **QN-2** as a photosensitizer, partial inhibition of the phototoxic effect was observed only in the presence of d-mannitol, suggesting that the Type II mechanism is mainly responsible for bacterial cell destruction. It is known that the intensity of the type I and type II mechanisms depends on several environmental factors (including oxygen availability).

### Cytotoxicity and Photobiocidal Properties

The compounds **QN-1**, **QN-2**, **QN-3**, **QD-1**, and **QD-2** can be considered as quaternary ammonium salts, and it was imperative to evaluate their biocidal efficacy against bacterial pathogens under dark conditions. For this purpose, the method of serial dilutions of these salts in an agar medium was used. Concentrations of compounds ranging from 1000 μg mL^–1^ to 31.25 μg mL^–1^ were examined against Gram-positive and Gram-negative bacterial strains. This study focused on three clinically significant microorganisms. *Staphylococcus aureus*, a notorious and ubiquitous pathogen, is responsible for a substantial and indeterminate incidence of simple skin infections, as well as an estimated hundreds of thousands to millions of more severe invasive infections worldwide annually.^27^ This bacterium is a predominant etiological agent in pneumonia and other respiratory tract infections, surgical site infections, prosthetic joint infections, cardiovascular infections, and nosocomial bacteremia. In particular, *S. aureus* is included in the World Health Organization’s 2024 list of bacterial priority pathogens (WHO BPPL), which requires heightened scrutiny due to its resistance to commercially available biocides.^28^

*Proteus mirabilis*, a Gram-negative bacterium exhibiting facultative anaerobic characteristics and rod-shaped morphology, is classified within the order *Enterobacterales* and the family *Enterobacteriaceae*. This organism is notable for its capacity to induce a variety of human infections, including those that affect wounds, ocular regions, the gastrointestinal tract, and the urinary system.^29^ *P. mirabilis* has been particularly implicated in catheter-associated urinary tract infections (CAUTIs) among the elderly population.^30^

The third pathogen examined in this study was *Acinetobacter baumannii*, a Gram-negative opportunistic pathogen that has demonstrated a marked increase in the infection rates in recent decades. The resistance of *A. baumannii* to standard antimicrobial therapies has been extensively documented, presenting a significant challenge in healthcare settings around the world.^31^ This species is classified as an ESKAPE pathogen, and carbapenem resistant *A. baumannii* has been designated as a top priority pathogen by the World Health Organization, highlighting the urgent need for the development of novel therapeutic strategies.^32^

The results of the biocidal activity of **QN-1**, **QN-2**, **QN-3**, **QD-1**, and **QD-2** are summarized in Table S6 (SI). It was established that the tested salts showed a negligible antibacterial activity. In most cases, the MIC value was 500 μg mL^–1^. Only compound **QN-2** inhibited the growth of *S. aureus* at a concentration of 62.5 μg mL^**–**1^. Subsequently, the maximum concentration of each compound that exhibits bactericidal activity at a level not exceeding 20% in the absence of light was established, and this parameter was designated as dark cytotoxicity.

As can be seen in Tables S7–S9 (SI), these salts showed a negligible dark cytotoxicity toward the studied bacterial strains, and it was considered that in all subsequent experiments of light-induced bactericidal activity these compounds would be used at a concentration of 2 μg mL^–1^. The photobactericidal activity of **QN-1**, **QN-2**, **QN-3**, **QD-1**, and **QD-2** was evaluated thereafter. The results indicated that compounds **QN-3** and **QD-2** demonstrated inadequate biocidal activity (Tables S1–S3; SI).

Accordingly, Figure 3, panel B illustrates the light-induced (energy fluence from 10 to 200 J·cm^–2^) biocidal activity exclusively for compounds **QN-1**, **QN-2**, and **QD-1**, which were identified as effective photosensitizers. The obtained results showed that in the case of **QN-1**, the biocidal activity was light-dose dependent, and *S. aureus* was killed particularly effectively. After just 2 min of exposure to light in the presence of this salt, a reduction in the number of live cells by 3.97 ± 0.3 log_10_ units was observed. After 5 min of irradiation (energy fluence of 50 J cm^–2^), the number of live cells was below the quantification level. Salts **QN-2** and **QD-1** were slightly less effective against this *coccus*, and a reduction in the number of live cells by 2.71 ± 0.3 and 3.61 ± 0.3 log_10_ units required just 10 min of irradiation (energy fluence of 100 J cm^–2^). A 15 min exposure to light (energy fluence of 150 J·cm^–2^) in the presence of these compounds resulted in extremely high cell mortality, and the number of live cells was below the quantification level.

The compounds **QN-1**, **QN-2**, and **QD-1** were also identified as effective photosensitizers for Gram-negative bacterial strains, specifically *P. mirabilis* and *A. baumannii*. As illustrated in Figure 3, panel B, the light-induced biocidal efficacy of these photosensitizing agents against the aforementioned bacterial rods was found to be contingent upon the light dosage, which is determined by the duration of the exposure. Notably, the optimal photokilling effect against *P. mirabilis* was observed in the presence of **QN-1**, where a 10–15 min irradiation period resulted in a reduction of viable cell counts by 3.79 to 3.92 log_10_ units (±0.2). No lethal effect (defined as the number of live cells below the quantification level) was recorded even after 20 min of blue light irradiation (light dose of 200 J·cm^–2^).

Furthermore, exposure of *A. baumannii* to light for 15 min in the presence of **QN-1** demonstrated a pronounced efficiency in photodynamic inactivation, with the surviving cell count falling below the detection threshold.

The photosensitizer **QN-2** exhibited the lowest efficacy in the photodynamic inactivation of the Gram-negative bacteria under the established exposure conditions. Following a 15 min irradiation period, the reduction in viable cell counts was quantified as 4.35 ± 0.2 log_10_ units for *P. mirabilis* and 3.96 ± 0.2 log_10_ units for *A. baumannii*. Conversely, the photosensitizer **QD-1** demonstrated the least effective photoinactivation of these bacterial rods, causing a reduction of only 2.1 ± 0.2 log_10_ units *for P. mirabilis* and 1.83 ± 0.2 log_10_ units for *A. baumannii* after the same duration of irradiation. These findings indicate a significant disparity in the efficacy of the tested photosensitizers in mediating photodynamic effects on the targeted bacterial species.

Our findings indicate a pronounced sensitivity of Gram-positive bacteria to light-induced eradication compared to Gram-negative pathogens, which corroborates with previous studies.^33^ It was hypothesized that this differential sensitivity arises from the unique structural characteristics of their cell envelopes.^34^ The enhanced susceptibility of *S. aureus* to antimicrobial photodynamic inactivation (aPDI) by the studied salts can be attributed to the unique structural characteristics of its cell wall, which is representative of Gram-positive bacteria. The cell wall of these *cocci* consists mainly of a highly cross-linked A3α-type peptidoglycan, featuring pentaglycine links that connect lysine in one muropeptide to the penultimate d-alanine of another. This peptidoglycan, along with ribitol-type teichoic acid chains linked to *N*-acetylmuramic acid residues, forms a multilayered structure around the *S. aureus* cell.^35^ The peptidoglycan layer is typically 20 to 30 nm thick, with short glycan strands (5 to 25 disaccharide units) and about 90% cross-linking.^36^

In contrast, Gram-negative bacteria possess a peptidoglycan layer that encases their cytoplasmic membrane, accompanied by an outer membrane (OM) that features an outer leaflet predominantly composed of lipopolysaccharide (LPS). The outer membrane acts as a strong barrier against chemical agents, enhancing the resistance of certain bacteria. It features an asymmetric lipid bilayer with phospholipids on the inner side and glycolipid lipopolysaccharides on the outer side.^37^ The OM contains two main types of proteins: transmembrane proteins, known as outer membrane proteins (OMPs), which typically have a β-barrel structure and lipoproteins that, although mostly soluble, have lipid components anchoring them to the OM. Unlike typical membranes that block proton passage, the OM allows small, water-soluble molecules like sugars and amino acids to diffuse freely. Additionally, the peptidoglycan layer in Gram-negative bacteria is generally thinner than that in Gram-positive bacteria, measuring less than 10 nm. Although both *Proteus* and *Acinetobacter* are categorized as Gram-negative bacteria, there is a paucity of detailed comparative studies regarding their cell wall composition and structural attributes in the current scientific literature. It is conceivable that differences may be present in the peptide cross-bridges of their peptidoglycan layers, as well as in the composition of their outer membrane proteins and lipopolysaccharides. These disparities may explain the observed variations in the susceptibility of these bacterial strains to photodynamic inactivation in the presence of the studied salts as photosensitizers. The explanation of this phenomenon requires further research, which has already begun in our laboratory.

The light-induced activity of compounds **QN-1**, **QN-2**, and **QD-1**, and the lack thereof for compounds **QN-0** and **QN-3** shed some light on the required molecular features of the sensitizer. The cationic fluorophore, the azoniabenzo­[*no*]­tetraphene ring system, was primary to the photosensitizing nature of the studied compounds. For achieving the photokilling of bacteria, there had to be a specific benzyl group at the quinolinium nitrogen atom as opposed to an *N*-methyl group. The compounds **QN-1** and **QN-3** differ in the steric bulk around the cationic center, where a larger group could result in more hydrophobic properties and less tight ion pair formation. Thus, the hydrophobic nature of the multiply fused ring system was necessary for achieving biological activity. Quaternization of the quinuclidine nitrogen atom suppresses charge transfer (CT) internally in the molecule from the locally excited aromatic ring systems, as evidenced in simple quinine derivatives.^38^ This effect appears to be responsible for a difference in the mechanism of photoactivation.^39^ The presence of a charged quinuclidinium also exerts a pronounced effect on the solubility, hydrophilicity, and ion pair formation. Particularly, **QN-2** is the most water-soluble among the tested compounds. In contrast, **QD-2**, despite having the same net molecular charge, is more compact due to conformational restrictions imposed by the quaternized bicyclic system. This likely promotes a tighter ion pair with its corresponding anion, possibly inhibiting longer-range electrostatic interactions. Experimentally, **QN-2** stands out, having the highest quantum fluorescence yield.

The monoquaternized pair **QN-1** and **QD-1** represent nearly mirror images, and their chemical properties are almost identical. In their antibacterial activity, usually the isomer of 7*R*,2′*S* configuration (**QN-1**, **QN-2**) was usually more effective than the 7*S*,2′*R* isomer (**QD-1, QD-2**). Their differential activity may stem from an at least partially specific interaction with a chiral cell component—proteins or, in the case of Gram-negative bacteria, LPS. The major contribution is the electrostatic interaction of the positively charged aromatic rings and the negative charges of carboxylate and phosphate residues. Otherwise, the compounds remain hydrophobic. Thus, the observed differences in light-induced biocidal activity are most likely the effect of differences in the localization and transport of the compounds to and from the cells. Overall, we believe that the microenvironment is responsible for the different modes of photoactivation rather than for the molecules alone.

The next phase of our experimental investigations focused on elucidating the interactions between the assessed photosensitizers and both Gram-positive and -negative bacterial cells. To achieve this objective, a variety of methodological approaches were utilized. The first one involved examining the changes in the ζ potential of bacterial cells under the influence of nontoxic concentrations of **QN-1**, **QN-2**, and **QD-1**. The surface charge of bacteria is often described by using the ζ potential, which is an electrochemical property of the cell surface. Halder et al.^40^ demonstrated that changes in the ζ potential can be associated with an improvement in the membrane permeability. The results obtained are summarized in Figure 4a. It was found that that in the absence of the studied salts, the bacterial cells had a negative potential (−22.3 ± 0.5 mV for *S. aureus*; −21.7 ± 0.3 mV for *P. mirabilis*; and −24.2 ± 0.3 mV for *A. baumannii*). Following incubation with **QN-1**, significant alterations in the surface charge were observed, with zeta potential values of −11.05 ± 0.1 mV for *S. aureus*, −12.9 ± 0.5 mV for *P. mirabilis*, and −12.5 ± 0.2 mV for *A. baumannii*. Treatment of these bacteria with **QN-2** resulted in a notable modification of the zeta potential, specifically for *A. baumannii*, which exhibited a value of −11.73 ± 0.5 mV. After incubation of bacterial cells with **QD-1**, insignificant changes in the ζ potential values were observed and the largest shift of this parameter was observed for *P. mirabilis* (−17.5 ± 0.5 mV).

**Figure 4:**
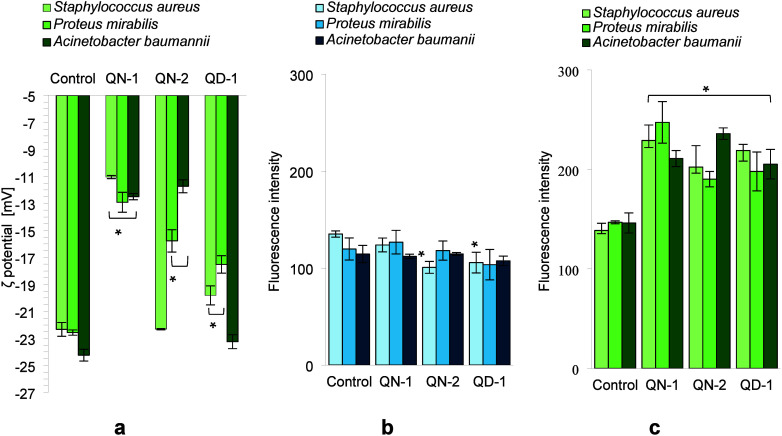
(a) Changes in the surface charge of bacterial cells after 1 h incubation with **QN-1**, **QN-2**, and **QD-1**; (b) changes in intracellular oxidative stress levels: after incubation bacterial cells with **QN-1**, **QN-2**, and **QD-1** (without light illumination); (c) after photodynamic inactivation of bacteria in the presence of **QN-1**, **QN-2**, and **QD-1**.

These results revealed that the most significant alterations in the surface charge of the examined bacteria were observed under the influence of **QN-1**, suggesting an increase in the permeability of the bacterial membranes. This phenomenon may be correlated to the enhanced phototoxic properties exhibited against the examined pathogens. On the contrary, **QD-1** induced the least alterations in the surface charge of the bacteria, which consequently resulted in the compound exhibiting weak photocidal properties.

On examination of the alterations in the zeta potential of the cell surface in response to **QN-1**, it can be suggested that this cationic molecule may interact with the surface structures of the cell envelopes, such as proteins and lipopolysaccharides (LPS). This interaction appears to influence the variations observed in the zeta potential.

The destabilizing properties of **QN-1**, **QN-2**, and **QD-1** toward the outer membrane of *P. mirabilis* and *A. baumannii* were also examined using the nondiffusible 1-*N*-phenylnaphthylamine (NPN) uptake assay, with EDTA serving as the reference control. It is well established that an intact outer membrane of bacteria functions as a permeability barrier, effectively excluding hydrophobic substances such as NPN. However, when the structural integrity is compromised, NPN is able to penetrate the phospholipid layer, resulting in a distinct increase in the fluorescence intensity. The results obtained are shown in Table S10 (SI). Based on these data, it is suggested that **QN-1**, **QN-2**, and **QD-1** do not demonstrate the ability to destabilize bacterial cell membranes.

The effect of **QN-1**, **QN-2**, and **QD-1** on changes in intracellular oxidative stress levels, determined using DCFH-DA, is presented in Figure 4b. The incubation of planktonic bacterial cells with **QN-1**, **QN-2**, and **QD-1** in the absence of light did not result in significant conversion of DCFH-DA to its fluorescent derivative, DCF (Figure 4b). The conversion process necessitated the subsequent illumination of the treated cells with blue light at a wavelength of 418 nm. The fluorescence intensity of DCF generated in the bacterial cells following one min of exposure to blue LED light, in the presence of **QN-1**, **QN-2**, and **QD-1**, exhibited a dependence on the specific bacterial strain. As depicted in Figure 4c, the most pronounced increase in DCF fluorescence intensity was observed following exposure of the studied strains to blue light in the presence of **QN-1**. The enhancement in fluorescence intensity ranged from 44% to 69% relative to the control group. These findings clearly indicated an increase in the amount of oxidizing compounds within the bacterial cells. The elevated levels of ROS in bacteria imply that the cell’s defense mechanisms are compromised, potentially leading to detrimental effects on the pathogen.

The cellular response of *S. aureus*, *P. mirabilis*, and *A. baumannii* to one minute of blue light irradiation in the presence of **QN-1**, **QN-2**, and **QD-1** as photosensitizers was determined by studying the effect of aPDI on the activity of superoxide dismutase (SOD), an important antioxidant enzyme. As can be seen in Table S11 (SI), the activity of superoxide dismutase after exposure of bacteria to light was not significantly higher compared to cells before bacteria treatment. The highest increase in the activity of this enzyme (approximately 20–23%) was detected in *S. aureus* and *P. mirabilis* cells that were exposed to LED light in the presence of **QN-1** and **QN-2** as photosensitizers, respectively.

The hemolytic activity of the examined salts on rabbit erythrocytes was assessed to evaluate the cytolytic properties of the tested compounds. As illustrated in Table S12 (SI), the extent of hemolysis in rabbit erythrocytes was found to be dependent on the concentration of the tested salts. At a concentration of 2 μg mL^–1^, the highest degree of erythrocyte membrane destruction, measured at 77.5 ± 2.4%, was observed with **QN-1**, in comparison to that of the control group treated with Triton X-100. Conversely, the lowest level of erythrocyte hemolysis, recorded at 42.1 ± 1.3%, was associated with the **QD-1** salt, following irradiation with blue light (418 nm). Generally, the hemolytic activity of the examined salts was found to be contingent on both their structural characteristics and concentration. Remarkably, the concentrations of these compounds were deemed nontoxic to bacteria under dark and used for photosensitization did not elicit significant hemolytic activity against rabbit erythrocytes.

It is pertinent to note that hemolysis is often used as an initial metric for toxicity evaluation.^41^

The fluorophore characteristics of the studied compounds indicate the potential for their accumulation within bacterial cells. The accumulation ability of the most photocidal salt (**QN-1**) was investigated by fluorescence microscopy. As shown in Figure 5, the cells of *S. aureus* exhibit intense fluorescence and are clearly visible as bright points. In contrast, for the examined Gram-negative rods, well-stained fluorescent cells are also evident; however, it is noteworthy that these bacterial cells form aggregates (Figures 5). These observations suggested that **QN-1** penetrates the cellular membranes of Gram-positive bacteria and accumulates within the cells of *S. aureus*. In the case of Gram-negative bacteria (*P. mirabilis* and *A. baumannii*), it appears that the compound remains largely outside the cells, leading to the formation of characteristic aggregates of these pathogens. A similar phenomenon was observed for **QN-2** and **QD-1** (Figure S5; SI).

**Figure 5:**
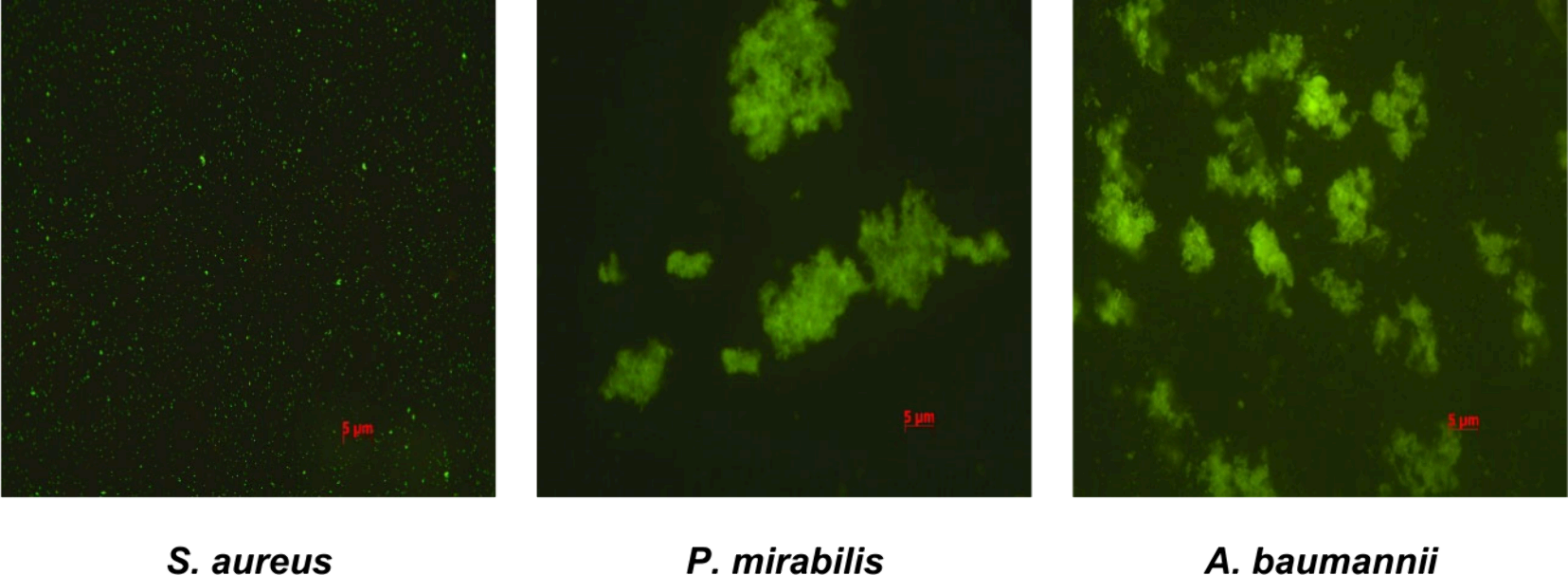
Microscopic image of fluorescent staining of bacteria using **QN-1**.

All of our findings highlight the potential of the studied molecules (particularly **QN-1**) for targeted antimicrobial photodynamic therapy, with efficacy influenced by the bacterial cell wall structure.

## Conclusions

In conclusion, our research highlights the promising potential of the synthesized azoniabenzo­[*no*]­tetraphenes derived from *Cinchona* alkaloids, particularly quinine and quinidine, as effective photosensitizers for antimicrobial photodynamic inactivation. The compounds demonstrated significant light absorption and fluorescence, with **QN-1**, **QN-2**, and **QD-1** exhibiting notable bactericidal effects, particularly against Gram-positive bacteria (*S. aureus*). This study also highlights the importance of structural characteristics in determining the efficacy of these salts, revealing that their phototoxic properties are influenced by factors such as membrane permeability and the generation of ROS. While the synthesized salts showed minimal dark toxicity, their effectiveness as photosensitizers was evident upon blue light (418 nm) exposure, with varying degrees of sensitivity observed among different bacterial strains. These findings pave the way for further studies on the optimization of these compounds, contributing to the development of novel therapeutic strategies to combat antibiotic-resistant pathogens and biofilms generated on abiotic surfaces. Future research will focus on elucidating the specific interactions between these compounds and bacterial cell structures, as well as enhancing their efficacy against a wider range of target microorganisms.

## Experimental Section

### General Experimental Procedures

All reagents were purchased from commercial suppliers. UV–vis absorption spectra were acquired on a Shimadzu UV-1650PC UV–vis spectrophotometer with a quartz cuvette. Fluorescence intensities were measured on a Spectra Max Gemini spectrofluorometer by using SoftMaxPro5 software. Fluorescent staining of cells was visualized under a EUROStar III Plus microscope (led light excitation, 460–490 nm).

Absorbance values were measured by using a BioTek Cytation Multimode Reader and Gen5 software. ^1^H and ^13^C NMR data were recorded on a Bruker Avance II 600 MHz spectrometer and referenced internally to TMS. Fluorescence emission spectra were recorded on a Horiba Fluoromax-4 instrument. The measurement of zeta potential was performed using a Zetasizer Ultra (Malvern Instruments, Worcesestershire, UK) and ZS XPLORER software.

### Microorganisms and Culture Conditions

In these studies, three bacterial species were used as test microorganisms: *Proteus mirabilis* (PCM 945), *Acinetobacter baumannii* (PCM8740), and *Staphylococcus aureus* (PCM2054). One colony of each bacteria was taken from an agar plate (Mueller-Hinton Agar, BTL, Poland) and was inoculated in 5 mL of Mueller–Hinton Broth (BTL, Poland). The prepared suspensions were incubated at 37 °C. After that time, 1 mL of each suspension was centrifuged separately (5 min/6000 rpm), the supernatant was harvested, and the pellet was resuspended in 1 mL of sterile deionized water. Bacterial suspensions were then adjusted to the McFarland 0.5 standard (OD_550_ = 0.09–0.1).

### Light Source

As a light source for photoinactivation, a single light-emitting diode (LED) with an output power of 58 mW (radiation intensity of 170 mW·cm^–2^) providing a wavelength of 418 nm was used.

### Fluorescence Quantum Yield Estimation

Sample compound solutions (10^–5^ M) in water–ethanol (1%) and a reference solution of quinine sulfate (4 × 10^–6^ M) in 0.5 M aqueous sulfuric acid were separately placed in a 1 cm fluorescence quartz cuvette. UV–vis spectra and fluorescence emission spectra excited at 350 and 415–438 nm were taken. Quantum fluorescence yields (φ_fl_) were estimated relative to quinine sulfate using the following formula:

1 $$\varphi_{\mathrm{fl}}=\varphi_{\mathrm{QN}}\frac{I_{\mathrm{sample}}\cdot A_{\mathrm{QN}\left(350\right)}}{A_{\mathrm{sample}}\cdot I_{\mathrm{QN}}}$$

where *φ*_QN_ = 0.546 is the fluorescence quantum yield for quinine sulfate,^3^ *A*_QN(350)_ is the absorbance of quinine sulfate solution at 350 nm, *I*_QN_ is the integral intensity of fluorescence of quinine sulfate solution, *A*_sample_ is the absorbance of sample solution at the excitation wavelength, and *I*_sample_ is the corresponding integral intensity of fluorescence.

### Photokinetic Studies – Photostability and Determination of the Type of Photosensitization Mechanism

The solutions of **QN-1**, **QN-2**, **QN-3**, **QD-1**, and **QD-2** were prepared by dissolving the compounds in DMSO and sterilizing by filtration through 0.22-μm pore diameter membranes (Millex-HP syringe-driven filter unit, Millipore). After filtration, the photosensitizer solutions were stored in the dark. The photostability of these photosensitizers was assessed by preparing 2 μg mL^–1^ solutions of tested compounds. The solution was then divided into two portions. The first was kept in the dark to serve as a control, and the second one was exposed to light (418 nm) for irradiation ranging from 2 to 20 min. The sample was placed in a cell made of quartz glass, and then irradiated for an appropriate amount of time. After irradiation, the absorbance of each sample was measured using a Shimadzu UV-1650PC UV–vis spectrophotometer, recording spectra from 300 to 600 nm. The absorbance of the irradiated samples was compared to the nonirradiated control.

Determination of the type of photosensitization mechanism was carried out using the following methods:

#### NBT (Nitro Blue Tetrazolium) Reduction - Type I Mechanism

Solutions (in DMSO) of the tested photosensitizers (2 μg mL^–1^) containing NADH (reduced nicotinamide adenine dinucleotide) at a concentration of 0.5 mM and NBT (nitro blue tetrazolium) at 0.2 mM were exposed to light (418 nm) for 2 to 25 min. The absorbance of the formed formazan was then measured at 560 nm by using a Shimadzu UV-1650PC UV–vis spectrophotometer. An unirradiated sample served as the control.

#### Tryptophan Photooxidation - Type II Mechanism

Solutions consisting of the salts tested at a concentration of 2 μg mL^–1^ and tryptophan at a concentration of 25 μM were irradiated with light of a wavelength of 418 nm in quartz cuvettes for 2 to 25 min. Tryptophan photooxidation was then determined (using a SpectraMax Gemini spectrofluorometer and SoftMaxPro5 software) on the basis of the decrease in fluorescence intensity at excitation and emission wavelengths of 290 and 343 nm, respectively. Samples without light exposure served as the control.

#### Singlet Oxygen Production - Type II Mechanism

To detect the ability of the tested compounds to produce singlet oxygen after irradiation, 9,10-anthracenediyl-bis­(methylene)­dimaleic acid sodium salt (ABDA) was used as the trapping agent. First, test solutions were prepared by mixing ABDA (final concentration of 0.15 mM) with the tested compounds (final concentrations of 2 μg·mL^–1^). The samples were then exposed to light (418 nm) for 1 to 25 min. Immediately after illumination, the absorbance was measured at 402 nm.

#### Singlet Oxygen Sensor Green

Singlet oxygen sensor green (SOSG) was used to test the ability of the studied compounds to generate singlet oxygen after light exposure. Stock solution of SOSG was prepared by dissolving 100 μg of the vial content provided by the supplier (Thermo Fisher Scientific) in 33 μL of methanol. The tested samples consisted of a mixture of one of the studied compounds together with SOSG at a final concentrations of 2 μg mL^–1^ and 5 μM, respectively. Then samples were exposed to light (418 nm) for 1 to 15 min. After each irradiation, the fluorescence intensity was measured at an excitation wavelength of 504 nm and an emission wavelength of 525 nm using a SpectraMax Gemini spectrofluorometer and the SoftMaxPro5 software. Samples not exposed to light served as the controls.

The inhibition of Type I mechanisms was assessed through the application of d-mannitol at a concentration of 50 mM, while the inhibition of type II mechanisms was investigated using sodium azide (NaN_3_) at an equivalent concentration of 50 mM, with both compounds acting as reactive oxygen species (ROS) suppressors. Initially, bacterial cells, at a density of 10^8^ CFU·mL^–1^, were incubated for a duration of 30 min with either d-mannitol or sodium azide at a noncytotoxic concentration, alongside a solution of the respective photosensitizer at a concentration of 2 μg·mL^–1^. Following incubation with **QN-1**, the bacterial cells underwent irradiation for 2 min; conversely, after incubation with **QN-2** and **QD-1**, cells were irradiated for 10 min utilizing a diode laser emitting at a wavelength of 418 nm. The efficacy of photodynamic inactivation employing d-mannitol and sodium azide was evaluated in accordance with the methodology using the drop plate technique.^42^

### Bacterial Inactivation Studies

#### Determination of MIC (Minimum Inhibitory Concentration)

A serial dilution method of the tested compounds on Mueller–Hinton Agar was used to determine the minimum inhibitory concentration (MIC). The studied salts were dissolved in DMSO and sterilized by using 0.22-μm pore diameter membranes (Millex-HP syringe-driven filter unit, Millipore). Briefly, 500 μL aliquots of liquid Mueller–Hinton Agar supplemented with the tested compounds were placed in glass tubes (final concentrations of compounds ranged from 1000 μg·mL^–1^ to 31.25 μg mL^–1^). Subsequently, 30 μL aliquots of the standardized suspension of bacteria were applied to the solid agar surface. The agar tubes were then incubated for 24 h at 37 °C, and after that time, an observation of the growth of microbial colonies on the surface of agar medium was conducted. The control consisted of agar tubes with the addition of dimethyl sulfoxide (DMSO) but without the tested salts. The MIC value was defined as the lowest concentration of a compound that under the experimental conditions inhibited the growth of colonies on the Mueller–Hinton Agar surface. This study was carried out in the same exact manner for each microorganism examined. All studies were performed in duplicate.

#### Dark Cytotoxicity Studies

270 μL of a standardized bacterial suspension were added to each well of a black sterile microtiter plate (Thermo Fisher Scientific). Then, 30 μL of the tested compounds (dissolved in dimethyl sulfoxide) were added to the wells to obtain a final compound concentration in the range of 2.0–8.0 μg·mL^–1^. The prepared suspensions were incubated for 30 min at 37 °C in the dark. The effect of the studied compounds on the viability of the microorganisms tested was evaluated using the drop plate technique.^42^

The reduction in viability of bacteria was calculated as

a) logarithmic reduction, according to the formula: *R* = log_10_ *N*_0_ – log _10_ *N*_0_;
b) percentage reduction, according to the formula: $R=\frac{\left(N_{0}-N\right)\times 100\%}{N_{0}}$ ;

where the number of bacteria before incubation with studied compounds is defined as *N*_0_, whereas *N* is the number of bacteria that remained alive after incubation with tested compounds. The experiments were carried out in triplicates.

#### Photodynamic Inactivation Studies

270 μL aliquots of the standardized bacterial suspension and 30 μL of tested compound (at a final concentration of 2 μg·mL^–1^) were added inside a black sterile microtiter plate and incubated for 30 min at 37 °C in the dark. Then, suspensions were exposed to light (λ = 418 nm) for 2, 5, 10, or 20 min. After irradiation, 100 μL of bacterial suspensions were harvested, and a series of dilutions were made. Each dilution was then seeded on Mueller–Hinton Agar using the drop plate technique.^42^ The suspensions without light irradiation served as the overall controls. The reduction in viability after photodynamic inactivation was calculated according to the equations described above. All experiments were carried out in triplicates.

#### Measurement of Zeta (ζ) Potential

The bacterial suspensions were prepared in a HEPES buffer solution (10 mM HEPES, 150 mM NaCl, pH 7.4) containing approximately 10^7^ CFU mL^–1^. Studied compounds were added into bacterial suspensions to obtain a final compound concentration of 2 μg mL^–1^. The untreated bacterial suspensions served as controls. The prepared samples were incubated for 1 h at 25 °C. After that time, suspensions were transferred into zeta cells and left for 2 min to equilibrate. The measurement were performed using Zetasizer Ultra (Malvern Instruments, Worcesestershire, UK) and ZS XPLORER software. The ζ potential was estimated using the mean of 20 readings. The waveform with an amplitude of 5 V was used for the determination of the ζ potential. Each experiment was repeated three times.

#### 1-*N*-Phenylnaphtylamine Probe Assay

To evaluate the uptake of *N*-phenyl-1-naphtylamine, a fluorescence-based assay was employed according to the procedure described by Hellander and Matilla-Sandholm with slight modifications.^43^ The standardized bacterial suspensions (see subsection Microorganisms and Culture Conditions) were prepared in 5 mM HEPES, and the examined salt was added to obtain a final concentration of 2 μg mL^–1^. Then, suspensions were incubated for 30 min at 37 °C on an incubator shaker (New Brunswick Scientific Innova40) at 121 rpm. Samples that were incubated without the tested compounds served as general controls. Following incubation, the samples were centrifuged at 6000 rpm for 5 min, supernatants were harvested, and the pellets were resuspended in 1 mL of 5 mM HEPES. Aliquots of 150 μL of the cell suspension were transferred to the wells of a white microtiter plate (Thermo Fisher Scientific). Each well received 50 μL of 160 μM NPN solution (prepared in 5 mM HEPES), and the samples were incubated for an additional 30 min. Subsequently, fluorescence intensities with an excitation wavelength of 350 nm and an emission wavelength of 400 nm were determined using a SpectraMax Gemini spectrofluorometer and the SoftMaxPro5 software. The experiments were conducted in triplicate.

#### Determination of the Oxidation Rate of Bacterial Cells

200 μL of standardized bacterial suspensions was treated with the solution of tested compounds and 2′,7′dichlorodihydrofluorescein diacetate (DCFH-DA) at a final concentration of 2 μg mL^–1^ and 50 μM, respectively. Then, the mixture was transferred to the wells of the black sterile microtiter plate and exposed to light for 1 min (418 nm). The production of reactive oxygen species (ROS) was measured using a spectrofluorometric technique, with the excitation set at 485 nm and emission at 520 nm, employing a Spectra Max Gemini spectrofluorometer and SoftMaxPro5 software. Each experiment was conducted in triplicate.

#### Superoxide Dismutase (SOD) Determination

To evaluate the activity of superoxide dismutase (SOD) in bacterial samples, an SOD determination kit purchased from Sigma-Aldrich was used, as well as the provided procedure. Briefly, standardized bacterial suspensions were incubated with tested compounds (final concentration was 2 μg mL^–1^) and irradiated for 1 min, as described in subsection Photodynamic Inactivation Studies. Then, reaction mixtures (Working Solution (WST); Enzyme Working Solution) were prepared according to the provided instruction. Twenty μL aliquots of each irradiated bacterial suspension were transferred to wells of the clear microtiter plate and mixed with 200 μL of WST and 20 μL of Enzyme Working Solution. At the same time, three blank samples were prepared. Each well of blank samples (Blank 1–3) consisted of 200 μL of WST. A 20 μL aliquots of deionized water were added to each Blank 1 and Blank 3 well. At the same time, 20 μL of the Enzyme Working Solution was added to each Blank 1 well, while 20 μL aliquots of dilution buffer were added to each Blank 3 well. Blank 2 served as a sample blank and was prepared by mixing 20 μL of tested sample with 200 μL of WST and 20 μL of dilution buffer (instead of Enzyme Working Solution). All samples and blanks were mixed thoroughly and incubated at 37 °C for 20 min. After that time, the absorbance of each well was measured at 450 nm using the BioTek Cytation Multimode Reader and Gen5 software. SOD activity was evaluated according the following equation:

$$\mathrm{SOD activity}\;\left(\%\right)=\frac{\left(A_{Blank1}-A_{Blank3}\right)-\left(A_{Sample}-A_{Blank2}\right)}{A_{Blank1}-A_{Blank3}}\times 100\%$$

where *A* is the absorbance of samples or blanks.

#### Determination of the Hemolytic Activity

The hemolytic properties of tested compounds were examined according to the standardized protocol proposed by Sæbø et al. with slight modifications.^41^ In brief, blood was collected and centrifuged at 6000 rpm for 5 min. Subsequently, supernatant was removed by aspiration, and erythrocytes were washed by adding 1 mL of PBS (phosphate-buffered saline, pH 7.0) and then centrifuged (6000 rpm/5 min). Washing was performed until the supernatant was clear. The supernatant was removed, and the pellet was suspended in 1 mL of PBS. Successively, 50 μL aliquots of the erythrocyte suspension were added to 950 μL of PBS with the tested compounds at the appropriate final concentrations. In parallel, control samples were prepared. The suspension of erythrocytes in PBS and the suspension of erythrocytes in PBS with 10% Triton-X-100 served as negative and positive controls, respectively. The samples were mixed thoroughly and allowed to incubate for 30 min at 37 °C. After that time, suspensions were centrifuged again (6000 rpm/5 min). 100 μL aliquots of the supernatant were transferred to a transparent microtiter 96-well plate (Thermo Fisher Scientific) and absorbance (*A*) values were measured at 405 nm using BioTek Cytation Multimode Reader and Gen5 software.

The hemolysis ratio (HR) was calculated using the following formula:

$$\mathrm{Hemolysis ratio}\;\left(\%\right)=\frac{A_{sample}-A_{negative}}{A_{positive}-A_{negative}}\times 100\%$$

where:

- *A*_sample_ is considered as absorbance of the sample containing studied compound;
- *A*_negative_ is considered as absorbance of the untreated sample (negative control);
- *A*_positive_ is considered as absorbance of the sample treated with Triton-X-100 (positive control).

All experiments were carried out in triplicates.

### Fluorescence Microscopy Studies

Fluorescent staining experiments with **QN-1** were performed using *S. aureus*, *P. mirabilis*, and *A. baumannii*. Briefly, 20 μL of the standardized bacterial suspension were placed on a microscopic slide previously coated with poly-d-lysine, and incubated for 30 min to enable the attachment of the cells to the glass slide. After that time, 10 μL of **QN-1** (final concentration of 2 μg mL^–1^) were placed on prepared slide and visualized under EUROStar III Plus microscope (led light excitation 460–490 nm).

### Statistical Analysis

Data are reported as mean ± standard deviation (SD) from triplicate experiments. The variation between two means was examined with a two-tailed unpaired Student *t* test. Statistical significance was determined at the *P*-values of <0.05.

## Supplementary Material



## Notes

Supporting Tables; S2. Photooxidation experiment; S3. Changes in SOSG fluorescence intensity following irradiation; S4. Microscopic images; S5. Experimental details for the synthesis of new compounds; S6. Plots of ^13^C and ^1^H NMR spectra; S7. HR-MS (ESI-TOF) for new compounds; S8. Supporting data for X-ray structure of QN-1 (PDF)
