# Supplementary material for: Quinine and Quinidine Derivatives as Photosensitizers for Photodynamic Inactivation of Bacterial Pathogens
Source: J Nat Prod. 2025 Aug 12;88(8):1907–18. doi: 10.1021/acs.jnatprod.5c00570 (PMC12379163; doi:10.1021/acs.jnatprod.5c00570)
Supplement: Supplementary file 1 [file np5c00570_si_001.pdf]

Supporting Information for:

Quinine and Quinidine Derivatives as Photosensitizers for Photodynamic Inactivation of Bacterial Pathogens

Irena Maliszewska,[a*] Anna Zdubek,[a] Błażej Dziuk,[b] Przemysław J. Boratyński[a]

[a]Department of Organic and Medicinal Chemistry, Faculty of Chemistry, Wrocław University of Science and Technology, Wybrzeże Wyspiańskiego 27, 50-370 Wrocław, Poland

[b] Institute of Advanced Materials, Faculty of Chemistry, Wrocław University of Science and Technology, Wybrzeże Wyspiańskiego 27, 50-370 Wrocław, Poland

*Correspondence: irena.helena.maliszewska@pwr.edu.pl

Table of Contents

S1. Supporting Tables....S-2
S2. Photooxidation experiment....S-5
S3. Changes in SOSG fluorescence intensity following irradiation....S-7
S4. Microscopic images....S-7
S5. Experimental details for the synthesis of new compounds....S-8
S6. Plots of ^{13}C and ^{1}H NMR spectra....S-13
S7. HR-MS (ESI-TOF) for new compounds....S-18
S8. Supporting data for X-ray structure of **QN-1**....S-20

S1. Supporting Tables

Table S1. The photobactericidal activity of **QN-3** and **QD-2** toward *S. aureus*.

Compound \ Time of irradiation	2 min	5 min	10 min	15 min	20 min
	**Cell viability [log$_{10}$CFU mL^{-1}]**				
**QN-3**	4.55 ± 0.11	lethal	-	-	-
**QD-2**	2.66 ± 0.31	lethal	-	-	-
**Control**	7.40 ± 0.03				

Table S2. The photobactericidal activity of **QN-3** and **QD-2** toward *P. mirabilis*.

Compound \ Time of irradiation	2 min	5 min	10 min	15 min	20 min
	**Cell viability [log$_{10}$CFU mL^{-1}]**				
**QN-3**	uncountable				
**QD-2**	7.42 ± 0.07	7.37 ± 0.03	7.16 ± 0.10	6.93 ± 0.07	-
**Control**	7.56 ± 0.05				

Table S3. The photobactericidal activity of **QN-3** and **QD-2** toward *A. baumannii*.

Compound \ Time of irradiation	2 min	5 min	10 min	15 min	20 min
	**Cell viability [log$_{10}$CFU mL^{-1}]**				
**QN-3**	7.33 ± 0.09	7.31 ± 0.07	7.28 ± 0.10	7.27 ± 0.13	7.28 ± 0.06
**QD-2**	7.24 ± 0.11	7.15 ± 0.06	7.14 ± 0.07	7.09 ± 0.10	-
**Control**	7.44 ± 0.05				

Table S4. The effect of NaN_3 on the viability of tested bacteria after exposure to light (418 nm).

Strain	**QN-1**		**QN-2**		**QD-1**	
	Without NaN_3	With NaN_3	Without NaN_3	With NaN_3	Without NaN_3	With NaN_3
	**Cell viability reduction (log$_{10}$ CFU mL^{-1})**					
S. aureus	3.97±0.03	3.26±0.03	2.71±0.03	2.93±0.03	3.61±0.03	3.53±0.03
A. baumannii	1.35±0.02	1.15±0.02	2.47±0.02	3.11±0.02	1.49±0.02	1.36±0.02

Table S5. Effect of D-mannitol on the viability of tested bacteria after exposure to light (418 nm).

Strain	**QN-1**		**QN-2**		**QD-1**	
	Without mannitol	With mannitol	Without mannitol	With mannitol	Without mannitol	With mannitol
	**Cell viability reduction (log_{10} CFU mL^{-1})**					
S. aureus	3.97±0.03	3.0±0.03	2.71±0.03	3.25±0.03	3.61±0.03	3.22±0.03
A. baumannii	1.35±0.2	1.32±0.02	2.47±0.2	1.37±0.02	1.49±0.2	1.34±0.02

Table S6. MIC values

	**QN-1**	**QN-2**	**QD-1**
	**MIC values [μg mL^{-1}]**		
S. aureus	500	62.5	250
P. mirabilis	500	500	250
A. baumannii	500	500	500

Table S7. The effect of tested compounds on the viability of *Staphylococcus aureus*; after 30 minutes of incubation estimated by colony counting assay (dark cytotoxicity)

**Compound** / **Concentration**	**8.0 μg mL^{-1}**	**4.0 μg mL^{-1}**	**2.0 μg mL^{-1}**
	**Cell viability [log_{10}CFUmL^{-1}]/[%]**		
**QN-1**	7.69 ± 0.05/ 73±8	7.73 ± 0.02/ 80±4	7.77 ± 0.03/ 87± 6
**QN-2**	7.47 ± 0.07/ 45±7	7.64 ± 0.05/ 65±8	7.75 ± 0.04/ 84±8
**QD-1**	7.28 ± 0.09/ 29±6	7.51 ± 0.06/ 48± 6	7.74 ± 0.05/ 81±10

Table S8. The effect of tested compounds on the viability of *Proteus mirabilis* after 30 minutes of incubation estimated by colony counting assay (dark cytotoxicity)

**Compound** / **Concentration**	**8.0 μg mL^{-1}**	**4.0 μg mL^{-1}**	**2.0 μg mL^{-1}**
	**Cell viability [log_{10}CFUmL^{-1}]/[%]**		
**QN-1**	7.92 ± 0.19/ 74± 30	7.98 ± 0.12/ 80±17	8.10 ± 0.10/ 105±26
**QN-2**	7.34 ± 0.04/ 66±7	7.29 ± 0.12/ 59±15	7.70 ± 0.19/ 159±55
**QD-1**	7.08 ± 0.13/ 71±19	7.18 ± 0.10/ 89±17	7.20 ± 0.11/ 94±23

Table S9. The effect of tested compounds on the viability of *Acinetobacter baumannii* after 30 minutes of incubation estimated by colony counting assay (dark cytotoxicity)

Compound \ Concentration	**8.0 µg mL^{-1}**	**4.0 µg mL^{-1}**	**2.0 µg mL^{-1}**
	**Cell viability [log$_{10}$CFUmL^{-1}]/[%]**		
**QN-1**	7.99 ± 0.13/ 210.53 ± 47.08	8.34 ± 0.05/ 457.90 ± 46.78	8.29 ± 0.13/ 421.05 ± 100.65
**QN-2**	8.01 ± 0.07/ 129.69 ± 22.10	7.97 ± 0.11/ 120.31 ± 31.29	7.97 ± 0.14/ 121.88 ± 43.17
**QD-1**	7.72 ± 0.03/ 78.32 ± 5.87	7.77 ± 0.03/ 87.66 ± 6.49	7.80 ± 0.03/ 93.64 ± 5.36

Table S10. The destabilizing properties of **QN-1**, **QN-2** and **QD-1** towards the outer membrane of *P. mirabilis* and *A. baumannii* examined using the non-diffusible 1-*N*-phenylnaphthylamine (NPN) uptake assay.

	**QN-1**	**QN-2**	**QD-1**	**Control**
	**Fluorescence intensity**			
P. mirabilis	468 ± 10	465 ± 15	594 ± 11	613 ± 33
A. baumannii	484 ± 32	524 ± 34	455 ± 20	573 ± 54

Table S11. Superoxide dismutase (SOD) activity detected in bacterial cells after aPDI

	**QN-1**	**QN-2**	**QD-1**
	**SOD activity [%]***		
S. aureus	123±8	97± 3	98±5
P. mirabilis	103±2	121± 1	103±2
A. baumannii	116±4	116±6	108±3

*SOD activity detected in cells before light irradiation was assumed as 100%

Table S12. The hemolysis ratio of rabbit erytrocytes after 30 minutes incubation with various concentrations of the studied salts

Concentration (µg mL^{-1})	**QN-1**	**QN-2**	**QD-1**
	**Hemolysis ratio (%)***		
2.0	78± 2	57± 2	42±1
4.0	81±5	103±4	105±3
8.0	97±5	119±6	124±7

S2. Photooxidation experiment

Photosensitization efficiency, i.e. the ability to generate photoreactive transient species (reactive oxygen species, ROS) of **QN-3** was further analyzed according to the procedure described below. A mixture containing 0.83 mg mL^{-1} of albumin and $1.3 \cdot 10^{-4}$ M of **QN-3** in phosphate-buffered saline (PBS) was placed in a quartz cuvette with frosted glass bottom. Light source was placed under the cuvette and irradiation with 418 nm light continued for 1-10 minute intervals. Successive fluorescence emission spectra were recorded with an excitation wavelength of 290 nm. Fluorescence in the 300-400 nm region was attributed to tryptophan residues in albumin, whereas fluorescence in the 450-700 nm region to compound **QN-3**. Artifact band $\lambda = 2 \times 290 \pm 5$ nm was not integrated (Figure S1). The photooxidation rate constant (kv) was calculated from semilogarithmic plots of the decrease in albumin fluorescence intensity versus irradiation time. The measurement is slightly perturbed by the buildup of a weak fluorescence band of λ_{max} at around 400 nm

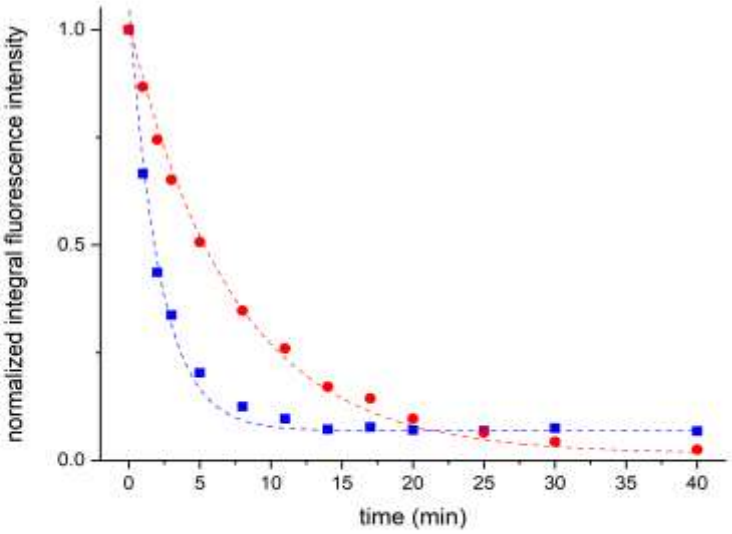

Figure S1. Decay of integral fluorescence at 300-400 nm (blue squares) and 450-700 nm (red circles) excited at 290 nm for 0.83 mg mL^{-1} albumin and $1.3 \cdot 10^{-4}$ M **QN-2** in PBS following irradiation from a 418 nm light source.

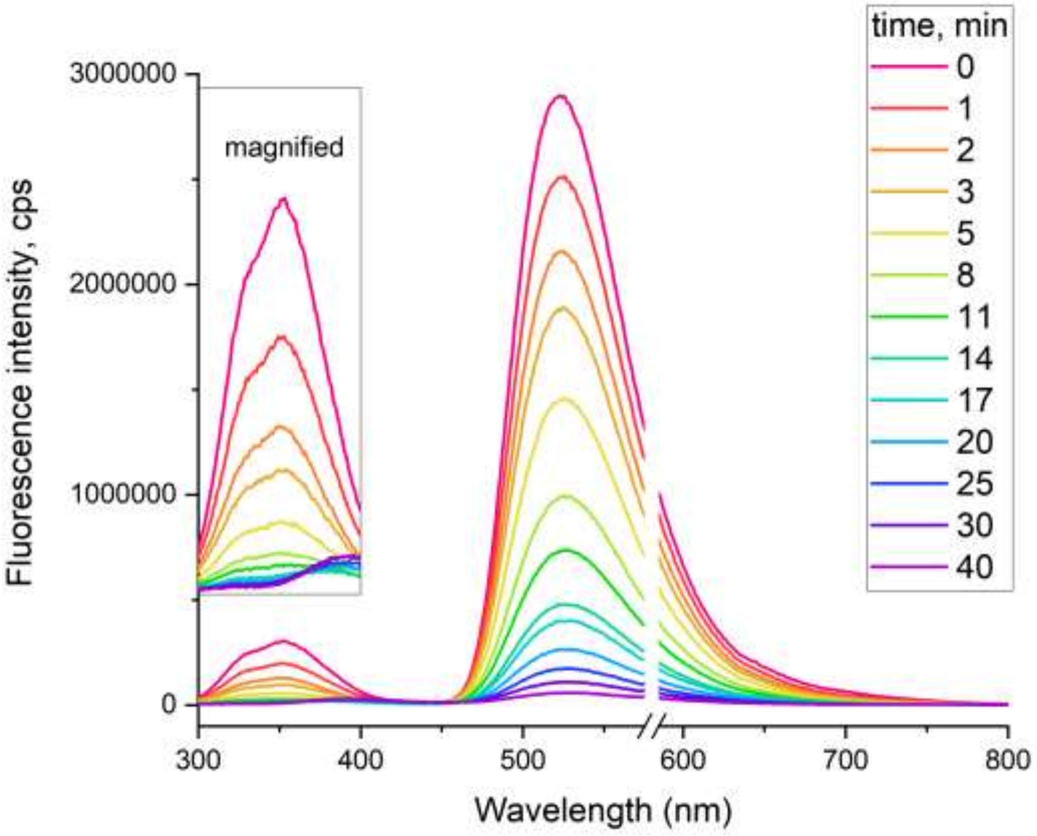

Figure S2. Successive emission fluorescence spectra (excitation wavelength 290 nm) for samples irradiated with 413 nm light for a cumulated time of 0-40 minutes. The inset plot shows a magnification of the 300-400 nm region. The artifact band corresponding to $\lambda_{EM} = 2 \times \lambda_{EX}$ at 575-585 nm is not shown.

S3. Changes in SOSG fluorescence intensity following irradiation

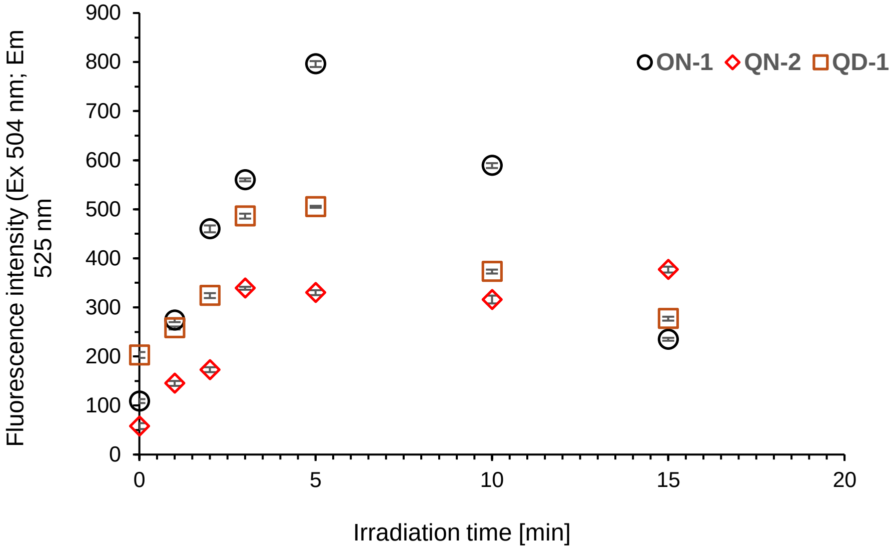

Figure S3. Changes in SOSG fluorescence intensity in the presence of **QN-1**, **QN-2**, and **QD-1.**

S4. Microscopic images

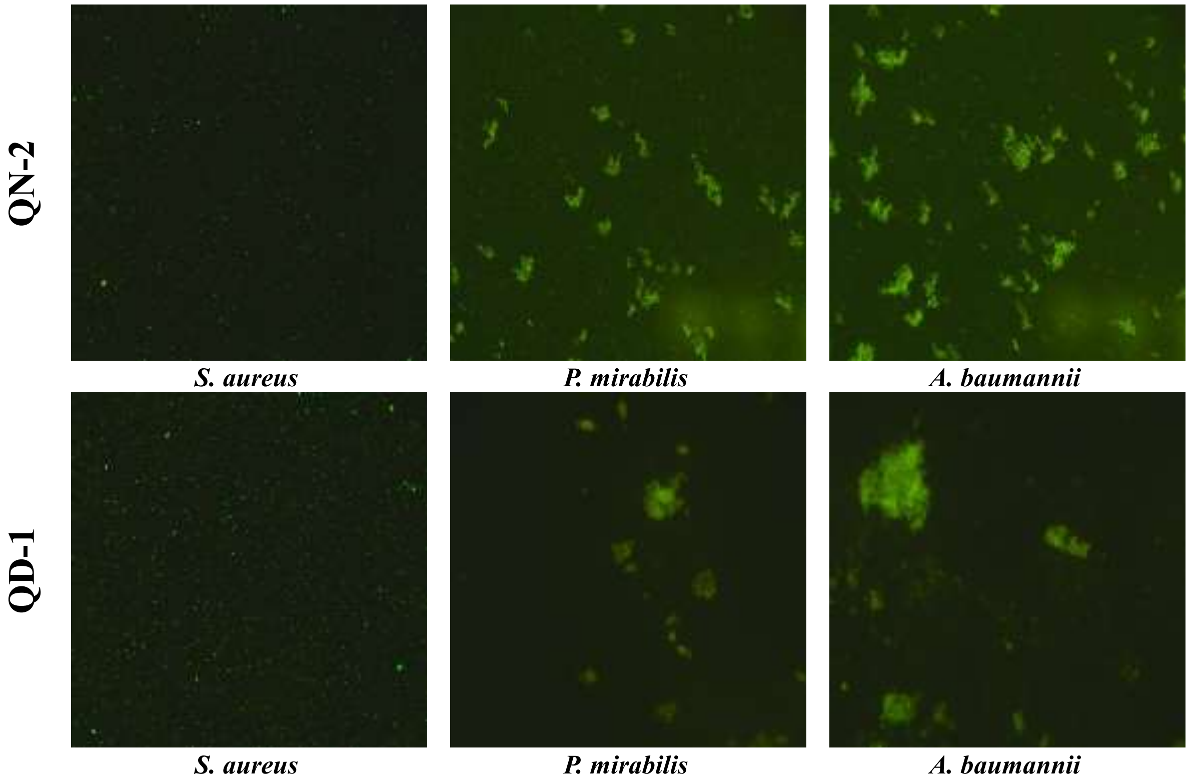

Figure S4. Microscopic images of fluorescent staining of bacteria using **QN-2** and **QD-1**. Magnification 200x.

S5. Experimental details for the synthesis of new compounds

All materials were purchased from commercial suppliers. *Cinchona*-alkaloid derived 5-azabenzo[*no*]tetraphenes (**QN-0**, **QD-0**) were obtained according to a reported procedure.[23]

General procedure for the quaternization. To a solution of 5-aza-2-methoxy-7-(5-vinylquinuclidin-2-yl)-7*H*-benzo[*no*]tetraphene (**QN-0** or **QD-0**, 256 mg, 0.59 mmol) in DMF was added alkyl halide (1-2.5 equiv). The mixture was briefly stirred and then stored at room temperature for 3-7 days. The solvent was partially removed at 40°C / 0.5 mmHg and the residue was suspended in acetone giving a yellow solid, which was washed with acetone. The solid was chromatographed on silica gel with CH_2Cl_2 / MeOH 20:1 to 9:1. Additional product crops could obtained by concentration of mother liquor and acetone extracts and subsequent chromatography on silica gel with DCM:MeOH 5-15%. The usual order of elution was: traces of unreacted starting material, mono quarternary salt, and di-quarternary salt.

2-((7*R*)-5-Azonia-5-benzyl-2-methoxy-7-((1*S*,2*S*,4*S*,5*R*)-5-vinylquinuclidin-2-yl)-7*H*-benzo[*no*]tetraphene bromide was obtained according to the general procedure from the starting material of quinine configuration using 1.05 equiv BnBr for 3 days in 69% yield as an orange amorphous solid. Sample crystalizes from DCM acetone and DMF:acetone.

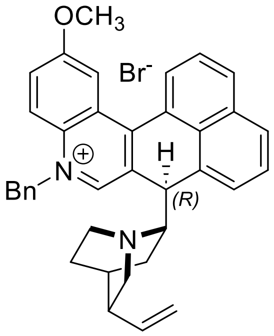

QN-1

^{1}H NMR (600 MHz, $CDCl_3$, TMS) δ 10.09 (s, 1H), 8.34-8.38 (m, 2H), 8.15 (d, J = 8.2 Hz, 1H), 7.89-7.92 (m, 2H), 7.71-7.74 (m, 2H), 7.64 (dd, J = 8.1, 7.1 Hz, 1H), 7.55 (d, J = 7.2 Hz, 2H), 7.50 (dd, J = 9.7, 2.8 Hz, 1H), 7.23-7.30 (m, 3H), 6.82 (br. d, J = 15.3 Hz, 1H), 6.38 (d, J = 15.3 Hz, 1H), 5.29 (ddd, J = 17.1, 10.3, 7.8 Hz, 1H), 4.83 (br., 1H), 4.73 (d, J = 17.1 Hz, 1H), 4.65 (d, J = 10.3 Hz, 1H), 3.94 (br., 1H), 3.82 (s, 3H), 2.93-3.05 (m, 2H), 2.13-2.25 (m, 3H), 1.81-1.87 (m, 1H), 1.69-1.72 (m, 1H), 1.54-1.57 (m, 1H), 1.21-1.28 (m, 1H),

0.78-0.85 (m, 1H) ppm. $^{13}C\{^1H\}$ NMR (151 MHz, $CDCl_3$, TMS) δ 159.4, 148.9, 144.3, 141.6, 133.9, 133.5, 133.2, 133.0, 132.5, 130.6, 130.1, 129.2, 129.0, 128.8, 128.2, 127.87, 127.80, 126.9, 125.5, 125.3, 123.9, 120.9, 114.4, 108.2, 62.4, 60.3, 56.3, 55.9, 45.3, 42.1, 40.5, 28.1, 27.9, 27.1 ppm. One sp^2 C signal not observed due to overlap. UV-Vis λ_{max} (logε): 272 (4.2), 342 (3.8), 436 nm (3.9). HRMS (ESI-TOF) *m*/*z* calcd. for $[C_{37}H_{37}N_2O]^+$ 523.2744, found: 523.2765.

(1*S*,2*S*,4*S*,5*R*)-2-((7*R*)-5-Azonia-5-benzyl-2-methoxy-7*H*-benzo[*no*]tetraphen-7-yl)-1-benzyl-5-vinylquinuclidin-1-ium dibromide was obtained according to the general procedure from the starting material of quinine configuration using 2.5 equiv BnBr for 4 days as deep orange film in 49% yield. With 1.2 equiv BnBr yield was 12%.

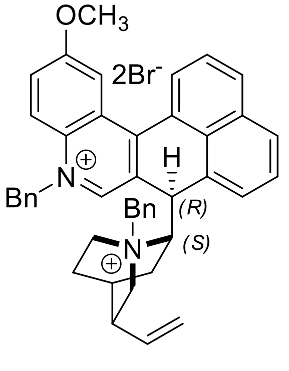

QN-2

^1H NMR (600 MHz, $CDCl_3$, TMS) δ 10.53 (s, 1H), 8.76 (d, *J* = 7.0 Hz, 1H), 8.61 (d, *J* = 7.4 Hz, 1H), 8.20 (d, *J* = 8.1 Hz, 1H), 8.18 (d, *J* = 9.5 Hz, 1H), 8.06-8.08 (m, 2H), 8.02 (d, *J* = 2.2 Hz, 1H), 7.97 (d, *J* = 8.2 Hz, 1H), 7.82-7.86 (m, 2H), 7.59 (dd, *J* = 9.6, 2.6 Hz, 1H), 7.45 (d, *J* = 7.4 Hz, 2H), 7.37-7.41 (m, 3H), 7.15-7.22 (m, 3H), 7.06 (br. s, 1H), 6.67 (d, *J* = 15.8 Hz, 1H), 6.25 (d, *J* = 15.8 Hz, 1H), 6.17 (d, *J* = 11.7 Hz, 1H), 5.93 (d, *J* = 11.7 Hz, 1H), 5.67 (ddd, *J* =17.2, 10.7, 6.8 Hz, 1H), 5.42-5.47 (m 1H), 5.21 (d, *J* = 17.2 Hz, 1H), 5.01 (d, *J* = 10.7 Hz, 1H), 4.38-4.42 (m, 1H), 4.11-4.18 (m, 1H), 3.98 (s, 3H), 3.15-3.21 (m, 1H), 3.15 (dd, *J* = 12.9, 10.9 Hz, 1H), 2.28-2.33 (m, 1H), 1.50-1.56 (m, 1H), 1.46-1.49 (m, 1H), 1.40-1.47 (m, 1H), 0.73-0.79 (m, 1H), 0.64-0.68 (m, 1H) ppm. $^{13}C\{^1H\}$ NMR (151 MHz, $CDCl_3$, TMS) δ 160.1, 148.2, 147.2, 135.8, 134.6, 133.7, 133.6, 132.9, 132.3, 131.2, 130.6, 130.24, 130.19, 129.9, 129.5, 129.1, 129.0, 128.9, 128.2, 128.1, 128.0, 127.6, 127.4, 126.1, 126.0, 125.4, 120.8, 118.3, 108.2, 72.4, 64.5, 62.0, 59.5, 56.1, 49.5, 41.7, 37.7, 26.7, 24.3, 23.7 ppm. UV-Vis λ_{max} (logε): 275 (4.2), 341 (3.7), 431 nm (4.0). HRMS (ESI-TOF) *m*/*z* calcd. for $[C_{44}H_{42}N_2O]^{2+}$ 307.1643, found: 307.1641.

2-((7*R*)-5-Azonia-5-methyl-2-methoxy-7-((1*S*,2*S*,4*S*,5*R*)-5-vinylquinuclidin-2-yl)-7*H*-

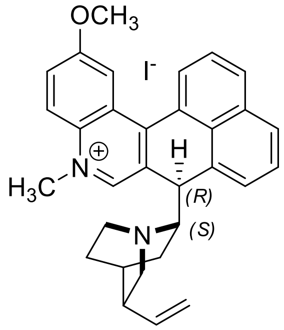

QN-3

benzo[*no*]tetraphene iodide was obtained according to the general procedure from the starting material of quinine configuration using 1.1 equiv iodomethane for 5 days as brown amorphous solid in 32% yield.

^{1}H NMR (600 MHz, $CDCl_3$+methanol-d_4 5:1, TMS) δ 9.12 (br. s, 1H), 8.60 (d, J = 7.3 Hz, 1H), 8.34 (d, J = 9.6 Hz, 1H), 8.26 (d, J = 8.1 Hz, 1H), 8.17 (d, J = 2.6 Hz, 1H), 7.98 (d, J = 8.1 Hz, 1H), 7.89 (t, J = 7.7 Hz, 1H), 7.82 (dd, J = 9.6, 2.6 Hz, 1H), 7.77 (br. d, J = 7.0 Hz, 1H), 7.67 (dd, J = 8.1, 7.0 Hz, 1H), 5.32 (ddd, J = 17.2, 10.4, 7.5 Hz, 1H), 4.77 (d, J = 17.2 Hz, 1H), 4.72 (d, J = 10.4 Hz, 1H), 4.7 (br., 1H) 4.67 (s, 3H), 4.05 (s, 3H), 3.65-3.73 (br., 1H), 2.98-3.11 (m, 2H), 2.21-2.45 (m, 3H), 1.86-1.92 (m, 1H), 1.75-1.78 (m, 1H), 1.62-1.68 (m, 1H), 1.24-1.28 (m, 1H), 0.79-0.89 (m, 1H) ppm. $^{13}C\{^1H\}$ NMR (151 MHz, $CDCl_3$+methanol-d_4 5:1, TMS) δ 160.3, 146.1, 145.8, 140.7, 134.1, 133.53, 133.44, 132.6, 130.0, 129.8, 129.4, 128.3, 127.8, 127.3, 126.8, 125.6, 125.3, 120.2, 114.5, 107.7, 62.0, 56.0, 55.9, 45.7, 45.4, 41.6, 39.6, 27.4, 27.3, 26.9 ppm(1 sp^2 carbon not observed due to coalescence or overlap). UV-Vis λ_{max} (logε): 270 (4.1), 340 (3.6), 430 nm (3.7). HRMS (ESI-TOF) *m*/*z* calcd. for $[C_{31}H_{31}N_2O]^+$ 447.2431, found: 447.2424.

2-((7*S*)-5-Azonia-5-benzyl-2-methoxy-7-((1*S*,2*R*,4*S*,5*R*)-5-vinylquinuclidin-2-yl)-7*H*-

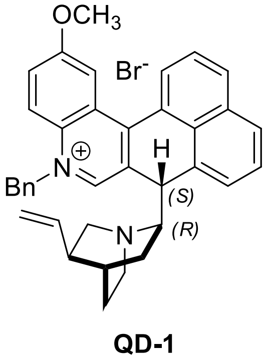

QD-1

benzo[*no*]tetraphene bromide was obtained according to the general procedure from the starting material of quinidine configuration using 1.2 equiv BnBr for 6 days in 58% yield as an orange amorphous solid.

^{1}H NMR (600 MHz, $CDCl_3$, TMS) δ 10.45 (s, 1H), 8.44 (d, J = 9.5 Hz, 1H), 8.33 (d, J = 7.4 Hz, 1H), 8.14 (d, J = 8.2 Hz, 1H), 7.90 (d, J = 8.1 Hz, 1H), 7.87 (d, J = 2.5 Hz, 1H), 7.73 (t, J = 7.8 Hz, 1H), 7.62-7.69 (m, 2H), 7.57 (d, J = 7.6 Hz, 2H), 7.50 (dd, J = 9.6, 2.6 Hz, 1H), 7.21-7.30 (m, 3H), 6.83 (br. d, J = 15.5 Hz, 1H), 6.50 (d, J = 15.5 Hz, 1H), 6.00 (ddd, J = 17.2, 10.6, 6.5 Hz, 1H), 5.32 (d, J = 17.2 Hz, 1H), 5.20 (d,

J = 10.6 Hz, 1H), 4.73 (br., 1H), 3.78 (s, 3H), 3.51-3.56 (m, 1H), 3.17 (dd, J = 14.0, 10.7 Hz, 1H), 2.67-2.73 (m, 1H), 2.34-2.40 (m, 1H), 2.27-2.32 (m, 1H), 2.18 (br., 1H), 1.64-1.67 (m, 1H), 1.44-1.49 (m, 1H), 1.37-1.42 (m, 1H), 1.17-1.24 (m, 1H), 0.50-0.55 (m, 1H) ppm. $^{13}C\{^1H\}$ NMR (151 MHz, $CDCl_3$, TMS) δ 159.4, 148.9, 144.2, 139.6, 133.9, 133.6, 133.1, 132.9, 132.4, 130.7, 130.1, 129.1, 129.0, 128.7, 128.1, 127.9, 127.6, 127.0, 126.88, 125.5, 125.3, 123.9, 121.1, 115.5, 108.1, 62.3, 60.2, 55.9, 49.4, 48.1, 45.0, 39.6, 28.2, 26.2, 26.1 ppm. UV-Vis λ_{max} (logε): 273 (4.3), 341 (3.7), 438 nm (4.1). HRMS (ESI-TOF) m/z calcd. for $[C_{37}H_{37}N_2O]^+$ 523.2744, HRMS found: 523.2765

(1*S*,2*R*,4*S*,5*R*)-2-((7*S*)-5-Azonia-5-benzyl-2-methoxy-7*H*-benzo[*no*]tetraphen-7-yl)-1-benzyl-5-vinylquinuclidin-1-ium dibromide was obtained according to the general procedure from the starting material of quinidine configuration using 1.2 equiv BnBr for 6 days in 12% yield as an orange amorphous solid.

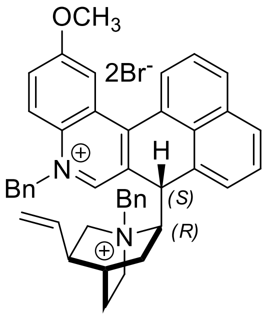

QD-2

^1H NMR (600 MHz, $CDCl_3$, TMS) δ 10.98 (s, 1H), 8.50-8.53 (m, 2H), 8.40 (d, J = 9.7 Hz, 1H), 8.19 (d, J = 8.2 Hz, 1H), 8.01 (d, J = 8.2 Hz, 1H), 7.91 (d, J = 2.6 Hz, 1H), 7.85-7.90 (m, 4H), 7.57-7.61 (m, 3H), 7.37-7.40 (m, 3H), 7.21-7.24 (m, 2H), 7.18 (t, J = 7.4 Hz, 1H), 6.82 (d, J = 2.2 Hz, 1H), 6.72 (d, J = 15.8 Hz, 1H), 6.43 (d, J = 15.8 Hz, 1H), 6.07 (d, J = 12.6 Hz, 1H), 5.48 (d, J = 12.6 Hz, 1H), 5.18-5.23 (m, 1H), 4.64-4.70 (m, 1H), 4.67 (d, J = 10.5 Hz, 1H), 4.52 (ddd, J = 17.2, 10.5, 6.1 Hz, 1H), 4.38 (d, J = 17.2 Hz, 1H), 3.94 (s, 3H), 3.31-3.36 (m, 1H), 2.77-2.82 (m, 1H), 2.58-2.64 (m, 1H), 2.09-2.17 (m, 2H), 1.53-1.61 (m, 3H), 0.91-0.98 (m, 1H) ppm. $^{13}C\{^1H\}$ NMR (151 MHz, $CDCl_3$, TMS) δ 160.1, 148.1, 147.7, 134.25, 134.21, 133.7, 133.4, 133.3, 133.18, 133.11, 130.7, 130.4, 130.2, 129.4, 129.2 (2C overlap), 128.9, 128.50, 128.47, 127.74, 127.71, 127.4, 127.2, 126.5, 126.3, 125.2, 121.5, 117.4, 108.2, 72.2, 65.0, 61.7, 57.4, 56.2, 53.1, 41.7, 37.3, 26.9, 23.3, 22.3 ppm. UV-Vis λ_{max} (logε): 274

(4.1), 340 (3.6), 440 nm (3.8). HRMS (ESI-TOF) *m/z* calcd. for $[C_{44}H_{42}N_2O]^{2+}$ 307.1643, found: 307.1641

S6. Plots of ^{13}C and ^{1}H NMR spectra

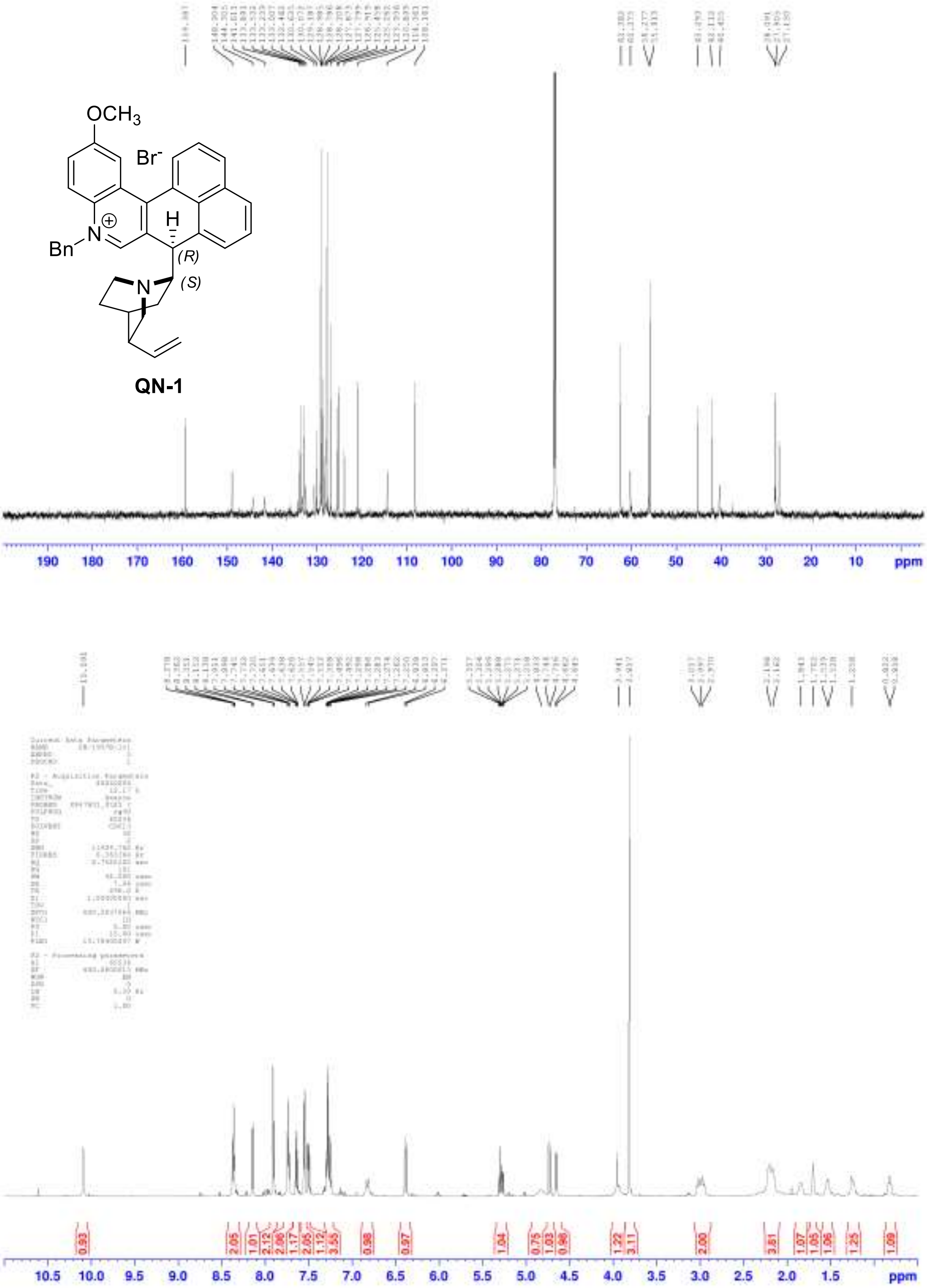

Figure S5. $^{13}C\{^{1}H\}$ NMR (151 MHz) and ^{1}H NMR (600 MHz) for **QN-1** in chloroform-*d*.

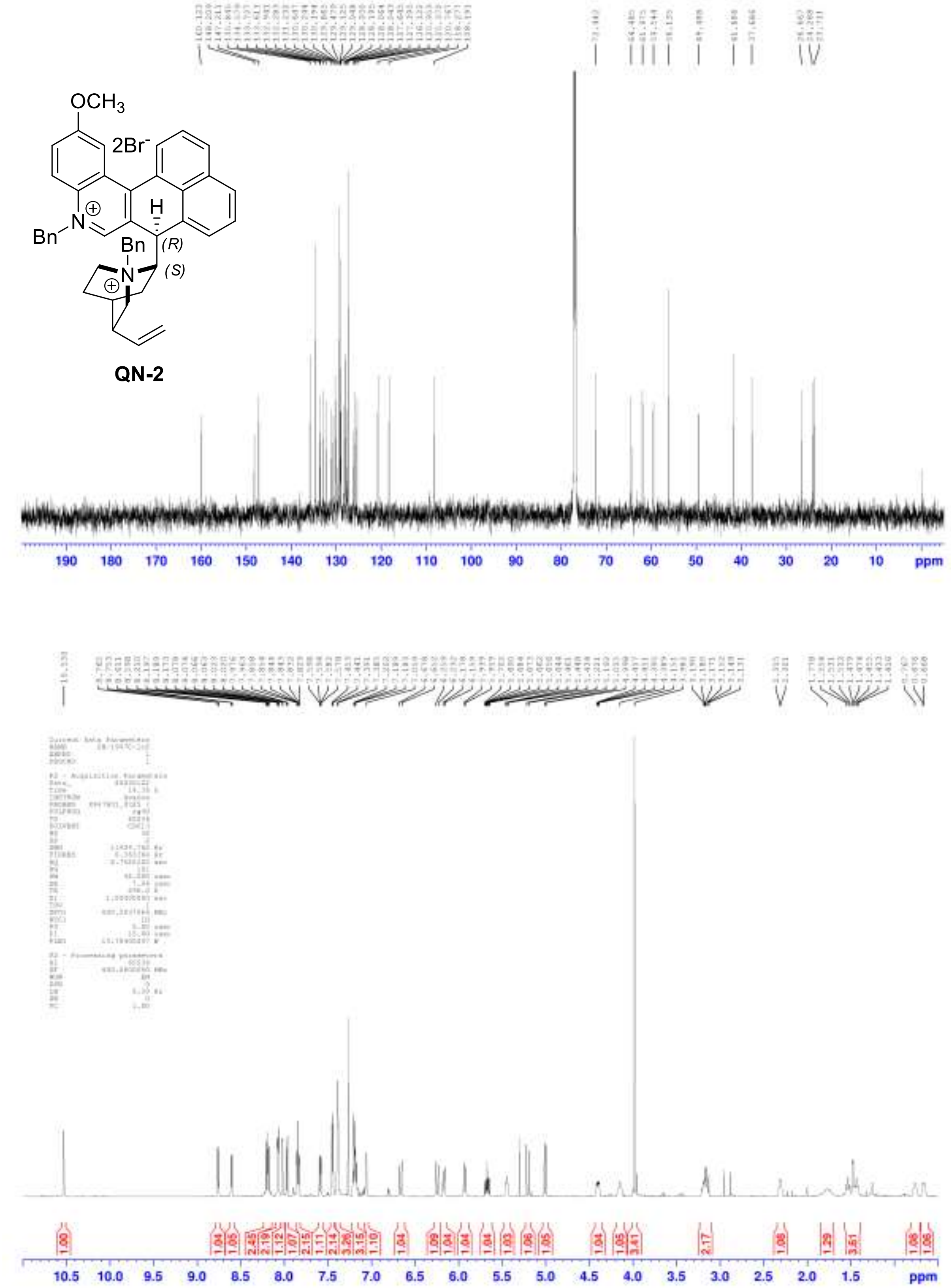

Figure S6. $^{13}C\{^{1}H\}$ NMR (151 MHz) and ^{1}H NMR (600 MHz) for **QN-2** in chloroform-*d*.

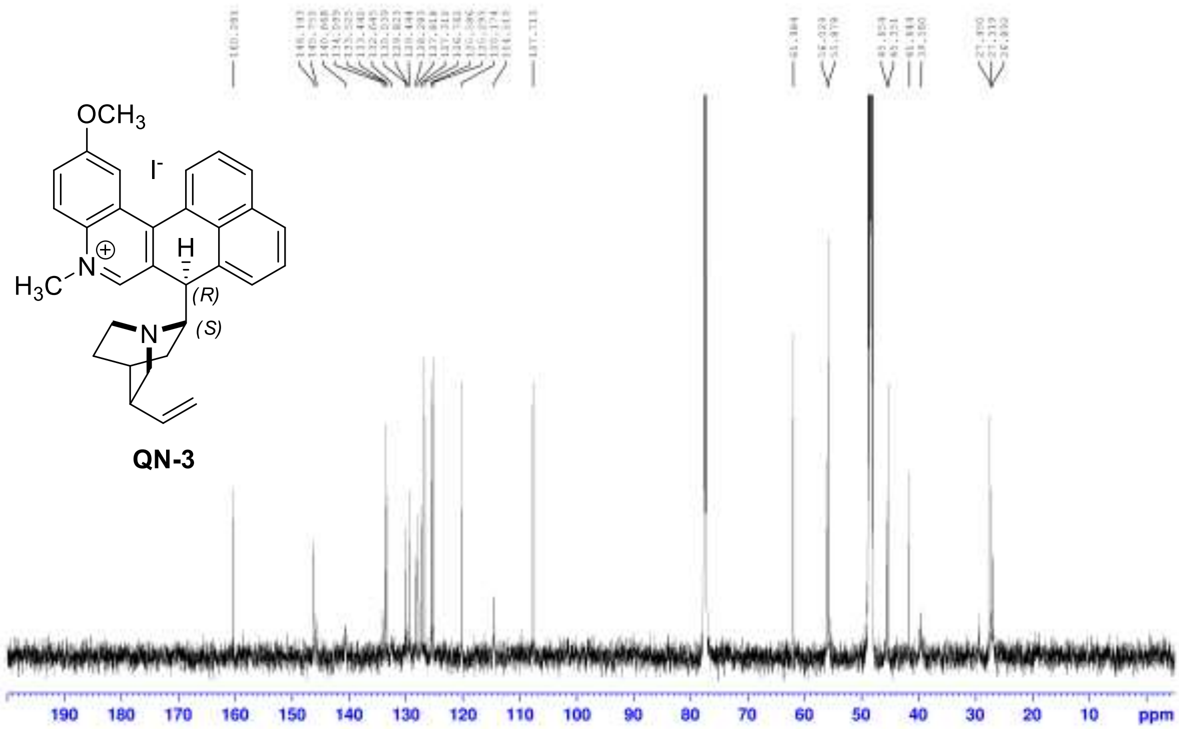

QN-3

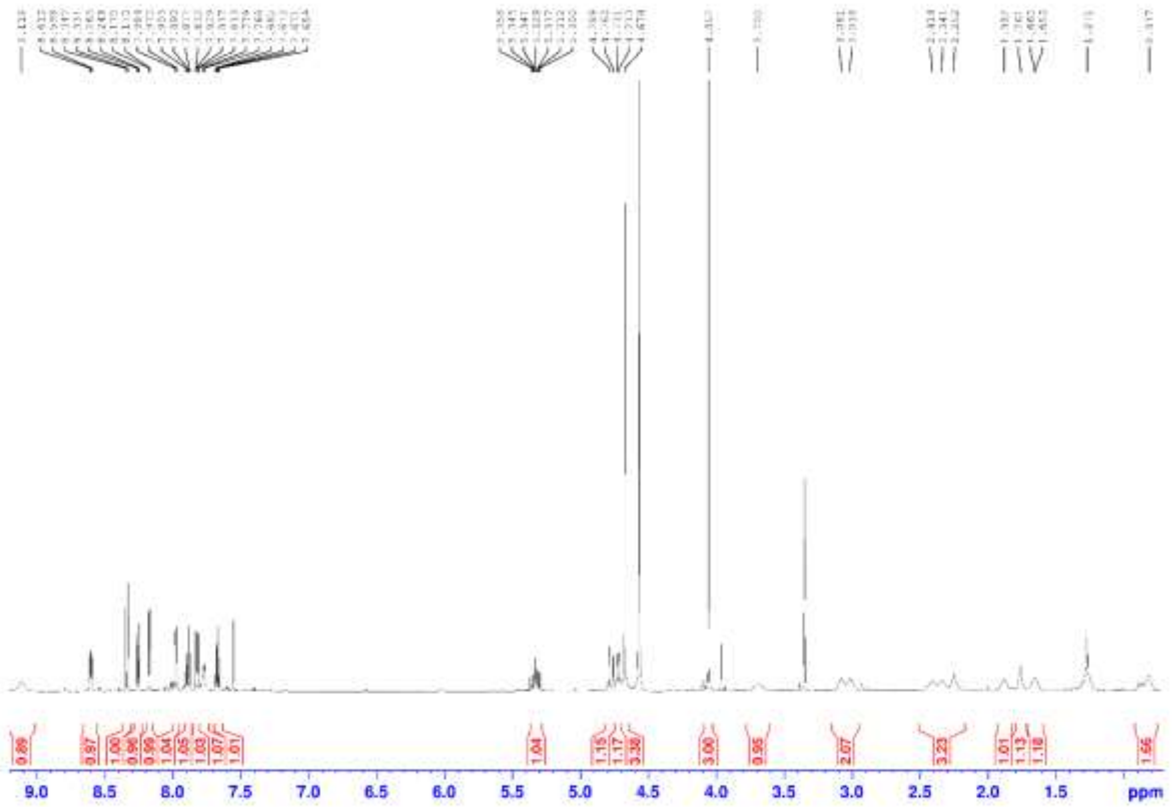

Figure S7. $^{13}C\{^{1}H\}$ NMR (151 MHz) and ^{1}H NMR (600 MHz) for **QN-3** in chloroform-*d* + methanol-d_4.

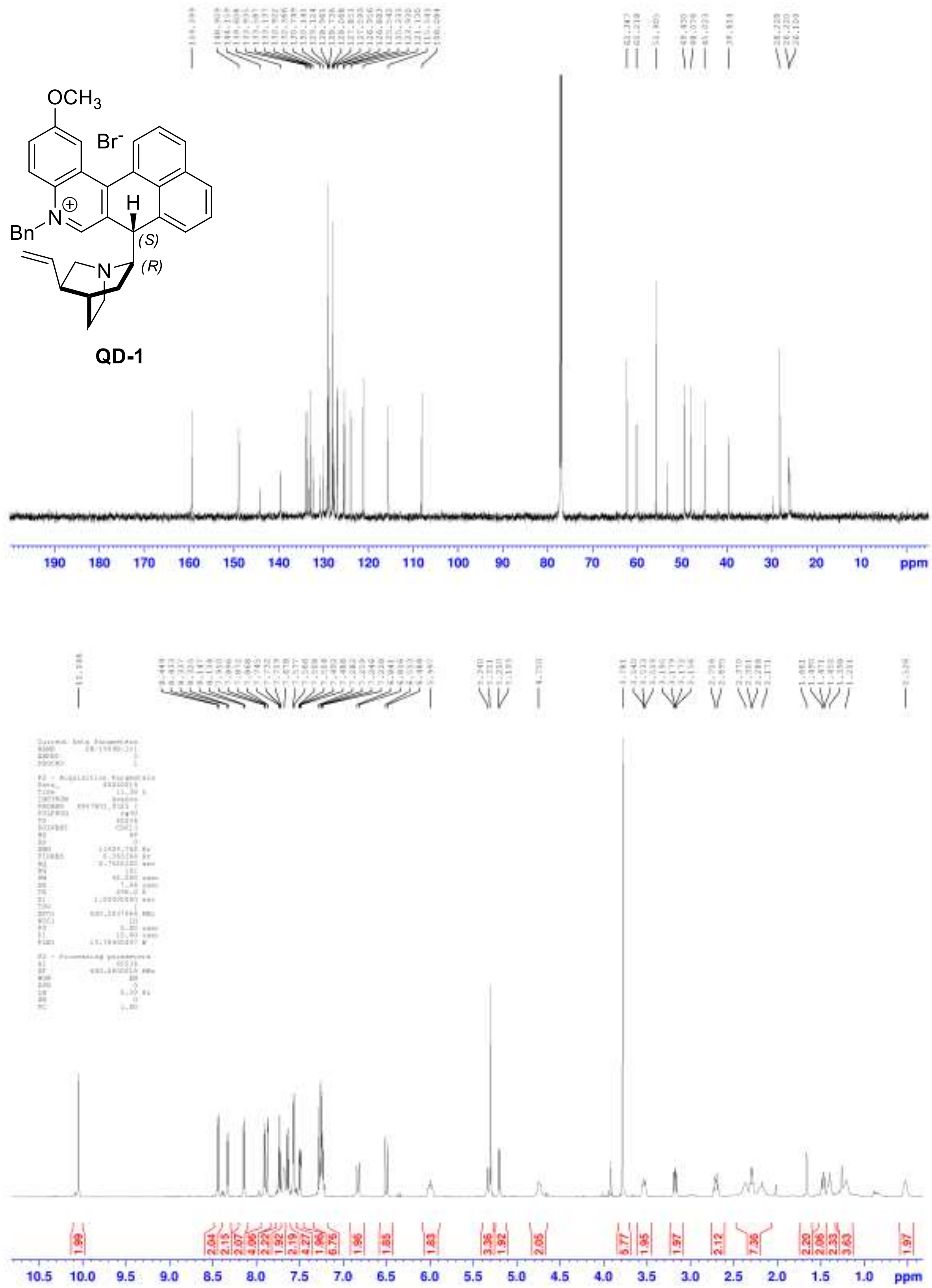

Figure S8. $^{13}C\{^{1}H\}$ NMR (151 MHz) and ^{1}H NMR (600 MHz) for **QD-1** in chloroform-*d*.

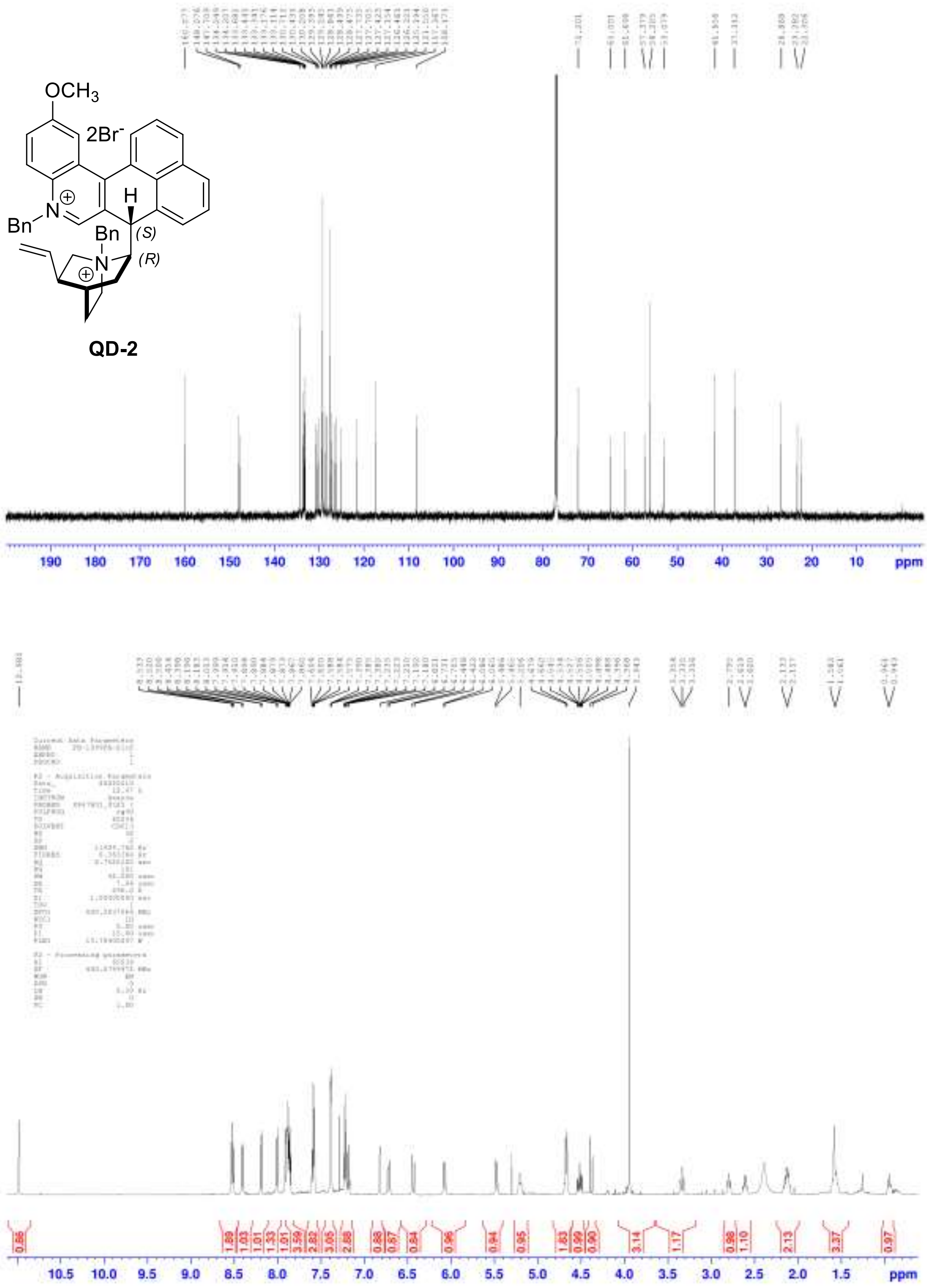

Figure S9. $^{13}C\{^{1}H\}$ NMR (151 MHz) and ^{1}H NMR (600 MHz) for **QD-2** in chloroform-*d*.

S7. HR-MS (ESI-TOF) for new compounds

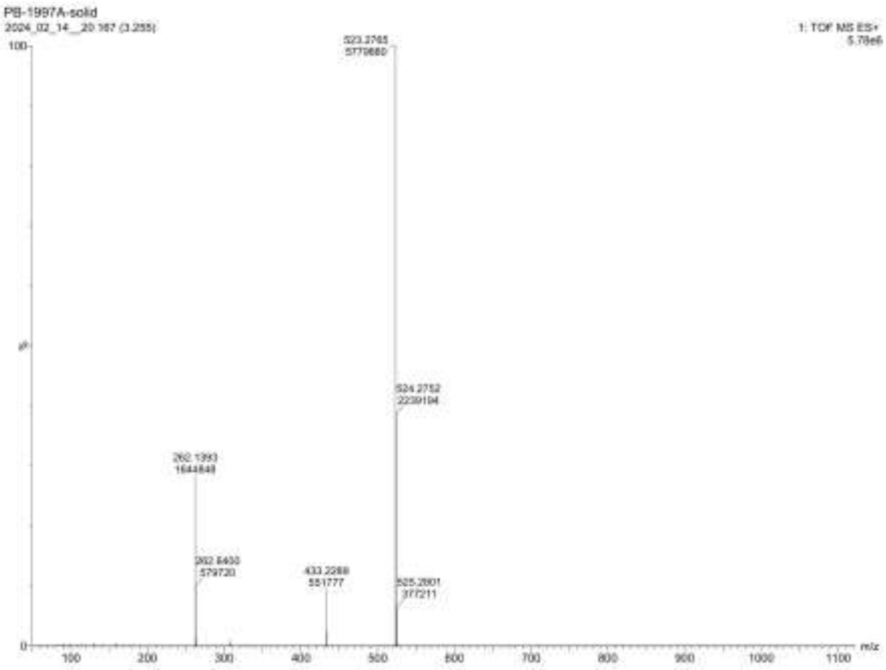

Figure S10. HR-MS (ESI-TOF) for **QN-1**

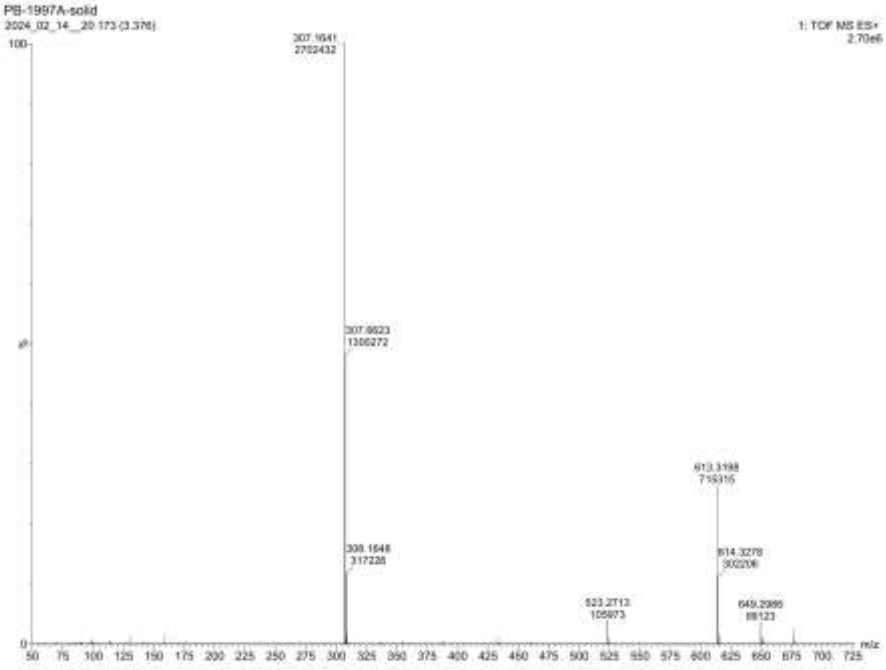

Figure S11. HR-MS (ESI-TOF) for **QN-2**

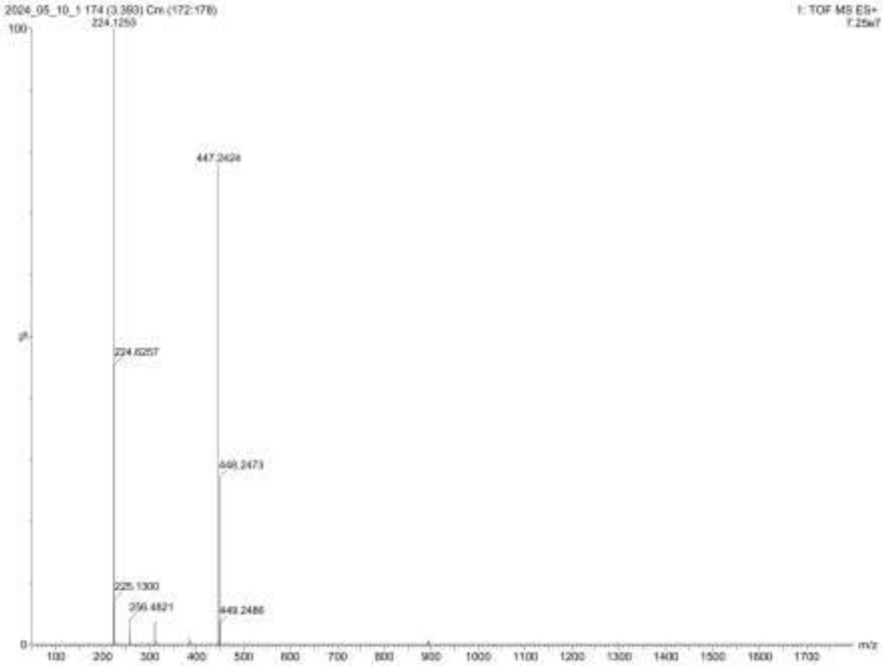

Figure S12. HR-MS (ESI-TOF) for **QN-3**

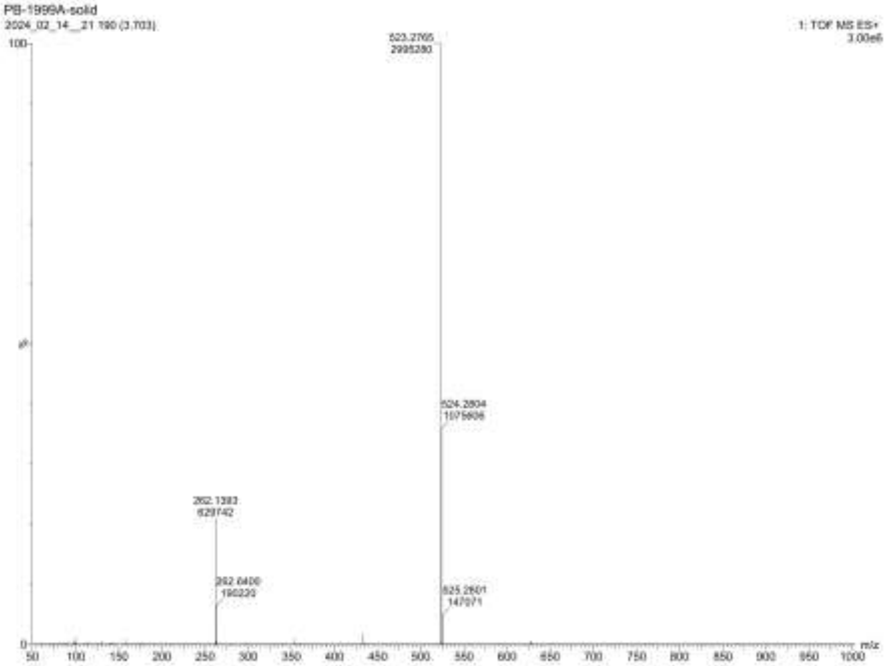

Figure S13. HR-MS (ESI-TOF) for **QD-1**

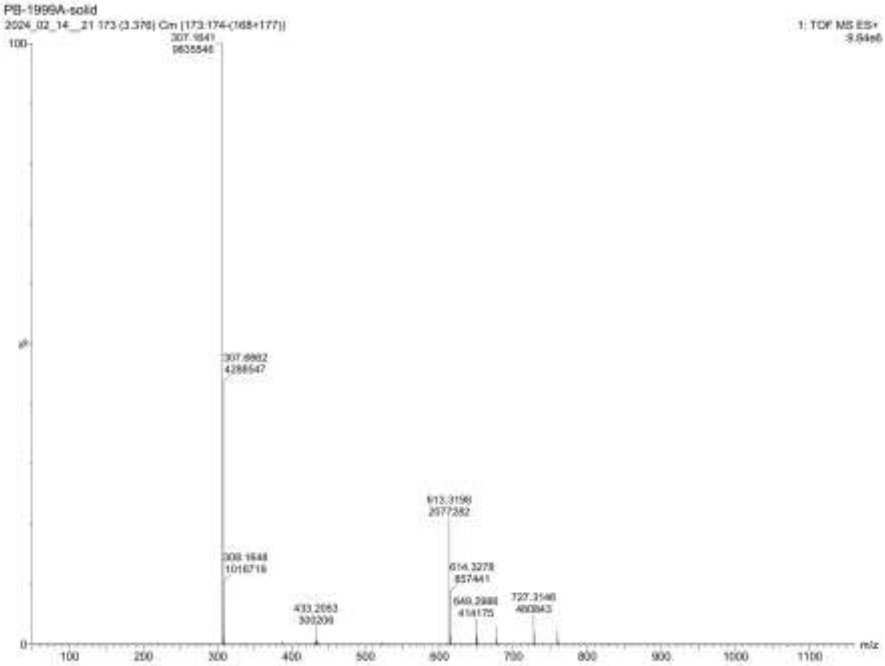

Figure S14. HR-MS (ESI-TOF) for **QD-2**

S8. Supporting data for X-ray structure of **QN-1**

The single crystal of **QN-1** was collected on a Rigaku Oxford Diffraction XtaLAB SynergyR DW diffractometer equipped with a HyPix ARC 150° Hybrid Photon Counting (HPC) detector using CuKα (λ = 1.54184 Å) at 100 K. The corrections to the Lorentz and polarization factors were applied to the reflection intensities.[S1] Data were processed using the CrysAlisPro software. The structures were solved by direct methods using SHELXS and refined by full-matrix least-squares methods based F^2 using SHELXL. [S2] The hydrogen atoms were determined from the geometric concepts and refined in a riding model with isotropic temperature factors of 1.2 times the Ueq value of the parent atom. All non-hydrogen atoms were located from difference Fourier

[S1] CrysAlis CCD; Oxford Diffraction Ltd: Abingdon, England, 2002. CrysAlis RED; Oxford Diffraction Ltd: Abingdon, England, 2002.

[S2] (a) G. M. Sheldrick, A short history of SHELX, Acta Crystallogr. Sect. A 64 (2008) 112−122, https://doi.org/10.1107/S0108767307043930. (b) G. M. Sheldrick, Crystal structure refinement with SHELXL, Acta Crystallogr. Sect. C 71 (2015) 3–8, https://doi.org/10.1107/S2053229614024218.

synthesis and refined by least squares method in the full-matrix anisotropic approximation. The crystallographic data for compounds and details of X-ray experiment are collected in Tables S8-S9. The structure drawings were prepared by using Mercury program.[S3] The coordinates of atoms and other parameters for structures were deposited with the Cambridge Crystallographic Data Centre: **2401157**; 12 Union Road, Cambridge CB2 1EZ, UK (Fax,_44-(1223)336-033, E-mail deposit@ccdc.cam.ac.uk).

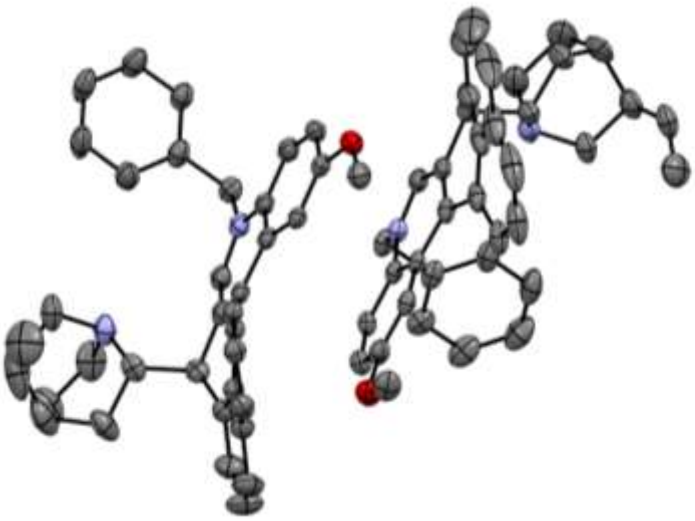

Figure S15. The molecular structure of compound **QN-1**. Hydrogen atoms and solvent molecules have been omitted for clarity.

[S3] C. F. Macrae, I. J. Bruno, J. A. Chisholm, P. R. Edgington, P. McCabe, E. Pidcock, L. Rodriguez-Monge, R. Taylor, J. van de Streek, P. A. Wood, New Features for the Visualization and Investigation of Crystal Structures, Journal of Applied Crystallography 41 (2008) 466–470, http://doi.org/10.1107/S0021889807067908.

Crystallographic data

Table S13. Experimental details for compound **QN-1**

Crystal data	
Chemical formula	Br·$C_{37}H_{35}N_2O$
M_r	603.58
Crystal system, space group	Orthorhombic, $P2_12_12_1$
Temperature (K)	100
a, *b*, *c* (Å)	11.9731 (1), 21.5323 (2), 24.3609 (1)
V ($Å^3$)	6280.44 (8)
Z	8
Radiation type	Cu *K*□
□ (mm^{-1})	2.01
Crystal size (mm)	0.1 × 0.08 × 0.05
Data collection	
Diffractometer	XtaLAB Synergy R, DW system, HyPix-Arc 150
Absorption correction	Multi-scan<br>*CrysAlis PRO* 1.171.43.105a (Rigaku Oxford Diffraction, 2024) Empirical absorption correction using spherical harmonics, implemented in SCALE3 ABSPACK scaling algorithm.
T_{min}, T_{max}	0.572, 1.000
No. of measured, independent and observed [$I > 2$□(I)] reflections	93297, 12876, 12238
R_{int}	0.029
(sin □/□)$_{max}$ ($Å^{-1}$)	0.628
Refinement	
$R[F^2 > 2$□$(F^2)]$, $wR(F^2)$, S	0.043, 0.110, 1.04
No. of reflections	12876
No. of parameters	749
H-atom treatment	H atoms treated by a mixture of independent and constrained refinement
□□$_{max}$, □□$_{min}$ (e $Å^{-3}$)	1.19, -1.30
Absolute structure	Flack x determined using 5134 quotients [(I+)-(I-)]/[(I+)+(I-)] (Parsons, Flack and Wagner, Acta Cryst. B69 (2013) 249-259).
Absolute structure parameter	-0.023 (3)

Table S14. Selected geometric parameters (Å, º).

O1B—C4B	1.359 (5)	C32B—C33B	1.402 (6)
O1B—C10B	1.423 (5)	C18A—H18A	0.9500
O1A—C4A	1.358 (5)	C18A—C17A	1.403 (7)
O1A—C10A	1.433 (5)	C26B—H26A	0.9900
N2B—C1B	1.389 (5)	C26B—H26B	0.9900
N2B—C9B	1.327 (5)	C26B—C25B	1.556 (6)
N2B—C31B	1.474 (5)	C17A—H17A	0.9500
N2A—C1A	1.391 (5)	C17A—C16A	1.371 (7)
N2A—C9A	1.331 (5)	C10B—H10A	0.9800
N2A—C31A	1.482 (5)	C10B—H10B	0.9800
C2B—H2B	0.9500	C10B—H10C	0.9800
C2B—C1B	1.419 (5)	C37A—H37A	0.9500
C2B—C3B	1.356 (5)	C37A—C32A	1.387 (6)
N1B—C22B	1.464 (6)	C37A—C36A	1.392 (6)
N1B—C26B	1.472 (5)	C32A—C33A	1.400 (6)
N1B—C27B	1.474 (6)	C36A—H36A	0.9500
C6B—C7B	1.437 (5)	C36A—C35A	1.373 (7)
C6B—C1B	1.415 (5)	C37B—H37B	0.9500
C6B—C5B	1.413 (5)	C37B—C36B	1.391 (6)
C7B—C8B	1.396 (6)	C36B—H36B	0.9500
C7B—C11B	1.472 (5)	C36B—C35B	1.391 (8)
N1A—C22A	1.477 (5)	C13B—H13B	0.9500
N1A—C26A	1.473 (5)	C13B—C14B	1.380 (10)
N1A—C27A	1.476 (6)	C29B—H29B	0.9500
C12A—H12A	0.9500	C29B—C25B	1.516 (7)
C12A—C13A	1.404 (5)	C29B—C30B	1.279 (8)
C12A—C11A	1.382 (5)	C35A—H35A	0.9500
C7A—C6A	1.436 (5)	C35A—C34A	1.372 (8)
C7A—C8A	1.391 (6)	C33B—H33B	0.9500
C7A—C11A	1.470 (5)	C33B—C34B	1.403 (6)
C8B—C9B	1.384 (6)	C16A—H16A	0.9500
C8B—C21B	1.510 (6)	C33A—H33A	0.9500
C3A—H3A	0.9500	C33A—C34A	1.379 (7)
C3A—C2A	1.354 (6)	C35B—H35B	0.9500
C3A—C4A	1.415 (6)	C35B—C34B	1.373 (8)
C4B—C3B	1.410 (5)	C10A—H10D	0.9800
C4B—C5B	1.370 (6)	C10A—H10E	0.9800
C6A—C1A	1.420 (5)	C10A—H10F	0.9800
C6A—C5A	1.415 (5)	C25A—H25A	1.0000
C1A—C2A	1.413 (6)	C25A—C26A	1.559 (8)
C5A—H5A	0.9500	C25A—C24A	1.527 (9)
C5A—C4A	1.363 (6)	C25A—C29A	1.521 (7)
C3B—H3B	0.9500	C23A—H23C	0.9900
C5B—H5B	0.9500	C23A—H23D	0.9900

C9A—H9A	0.9500	C23A—C24A	1.533 (7)
C9A—C8A	1.395 (5)	C25B—H25B	1.0000
C2A—H2A	0.9500	C25B—C24B	1.527 (8)
C21A—H21A	1.0000	C14B—H14B	0.9500
C21A—C8A	1.504 (6)	C14B—C15B	1.399 (10)
C21A—C22A	1.553 (6)	C34B—H34B	0.9500
C21A—C19A	1.509 (6)	C26A—H26C	0.9900
C13A—H13A	0.9500	C26A—H26D	0.9900
C13A—C14A	1.361 (6)	C15B—C16B	1.471 (10)
C11A—C20A	1.440 (5)	C24A—H24A	1.0000
C11B—C12B	1.387 (6)	C24A—C28A	1.511 (7)
C11B—C20B	1.432 (7)	C34A—H34A	0.9500
C12B—H12B	0.9500	C27B—H27A	0.9900
C12B—C13B	1.401 (6)	C27B—H27B	0.9900
C9B—H9B	0.9500	C27B—C28B	1.559 (8)
C22A—H22A	1.0000	C30B—H30A	0.95 (5)
C22A—C23A	1.540 (6)	C30B—H30B	0.98 (7)
C20B—C19B	1.417 (8)	C23B—H23A	0.9900
C20B—C15B	1.418 (7)	C23B—H23B	0.9900
C19A—C20A	1.424 (6)	C23B—C24B	1.541 (8)
C19A—C18A	1.370 (6)	C24B—H24B	1.0000
C14A—H14A	0.9500	C24B—C28B	1.536 (9)
C14A—C15A	1.411 (6)	C18B—H18B	0.9500
C31B—H31A	0.9900	C18B—C17B	1.389 (10)
C31B—H31B	0.9900	C29A—H29A	0.9500
C31B—C32B	1.522 (5)	C29A—C30A	1.194 (10)
C22B—H22B	1.0000	C27A—H27C	0.9900
C22B—C21B	1.564 (5)	C27A—H27D	0.9900
C22B—C23B	1.549 (6)	C27A—C28A	1.533 (8)
C21B—H21B	1.0000	C16B—H16B	0.9500
C21B—C19B	1.493 (7)	C16B—C17B	1.337 (12)
C20A—C15A	1.406 (6)	C28A—H28C	0.9900
C31A—H31C	0.9900	C28A—H28D	0.9900
C31A—H31D	0.9900	C28B—H28A	0.9900
C31A—C32A	1.515 (6)	C28B—H28B	0.9900
C15A—C16A	1.419 (6)	C17B—H17B	0.9500
C19B—C18B	1.371 (7)	C30A—H30C	0.9500
C32B—C37B	1.386 (6)	C30A—H30D	0.9500
C4B—O1B—C10B	118.4 (3)	C25B—C26B—H26B	109.4
C4A—O1A—C10A	117.9 (3)	C18A—C17A—H17A	119.6
C1B—N2B—C31B	121.6 (3)	C16A—C17A—C18A	120.8 (4)
C9B—N2B—C1B	120.0 (3)	C16A—C17A—H17A	119.6
C9B—N2B—C31B	118.1 (3)	O1B—C10B—H10A	109.5
C1A—N2A—C31A	121.9 (3)	O1B—C10B—H10B	109.5
C9A—N2A—C1A	120.9 (3)	O1B—C10B—H10C	109.5

C9A—N2A—C31A	117.2 (3)	H10A—C10B—H10B	109.5
C1B—C2B—H2B	120.2	H10A—C10B—H10C	109.5
C3B—C2B—H2B	120.2	H10B—C10B—H10C	109.5
C3B—C2B—C1B	119.6 (3)	C32A—C37A—H37A	119.9
C22B—N1B—C26B	107.5 (3)	C32A—C37A—C36A	120.2 (4)
C22B—N1B—C27B	111.8 (4)	C36A—C37A—H37A	119.9
C26B—N1B—C27B	107.7 (4)	C37A—C32A—C31A	119.7 (4)
C1B—C6B—C7B	119.0 (3)	C37A—C32A—C33A	118.8 (4)
C5B—C6B—C7B	123.6 (3)	C33A—C32A—C31A	121.5 (4)
C5B—C6B—C1B	117.4 (3)	C37A—C36A—H36A	119.8
C6B—C7B—C11B	124.5 (4)	C35A—C36A—C37A	120.4 (5)
C8B—C7B—C6B	117.4 (3)	C35A—C36A—H36A	119.8
C8B—C7B—C11B	118.0 (4)	C32B—C37B—H37B	119.9
C26A—N1A—C22A	106.5 (3)	C32B—C37B—C36B	120.3 (4)
C26A—N1A—C27A	108.2 (4)	C36B—C37B—H37B	119.9
C27A—N1A—C22A	110.1 (4)	C37B—C36B—H36B	120.1
N2B—C1B—C2B	119.6 (3)	C35B—C36B—C37B	119.8 (5)
N2B—C1B—C6B	119.7 (3)	C35B—C36B—H36B	120.1
C6B—C1B—C2B	120.6 (3)	C12B—C13B—H13B	119.7
C13A—C12A—H12A	119.3	C14B—C13B—C12B	120.5 (6)
C11A—C12A—H12A	119.3	C14B—C13B—H13B	119.7
C11A—C12A—C13A	121.4 (4)	C25B—C29B—H29B	115.5
C6A—C7A—C11A	125.0 (4)	C30B—C29B—H29B	115.5
C8A—C7A—C6A	117.4 (4)	C30B—C29B—C25B	129.1 (5)
C8A—C7A—C11A	117.5 (3)	C36A—C35A—H35A	120.1
C7B—C8B—C21B	121.8 (4)	C34A—C35A—C36A	119.7 (4)
C9B—C8B—C7B	120.0 (4)	C34A—C35A—H35A	120.1
C9B—C8B—C21B	118.0 (4)	C32B—C33B—H33B	120.6
C2A—C3A—H3A	119.6	C32B—C33B—C34B	118.8 (5)
C2A—C3A—C4A	120.8 (4)	C34B—C33B—H33B	120.6
C4A—C3A—H3A	119.6	C15A—C16A—H16A	119.9
O1B—C4B—C3B	115.2 (4)	C17A—C16A—C15A	120.2 (4)
O1B—C4B—C5B	124.6 (4)	C17A—C16A—H16A	119.9
C5B—C4B—C3B	120.1 (4)	C32A—C33A—H33A	120.0
C1A—C6A—C7A	119.6 (4)	C34A—C33A—C32A	120.0 (4)
C5A—C6A—C7A	123.0 (4)	C34A—C33A—H33A	120.0
C5A—C6A—C1A	117.4 (4)	C36B—C35B—H35B	119.9
N2A—C1A—C6A	118.9 (4)	C34B—C35B—C36B	120.3 (4)
N2A—C1A—C2A	120.5 (3)	C34B—C35B—H35B	119.9
C2A—C1A—C6A	120.6 (4)	O1A—C10A—H10D	109.5
C6A—C5A—H5A	119.3	O1A—C10A—H10E	109.5
C4A—C5A—C6A	121.3 (4)	O1A—C10A—H10F	109.5
C4A—C5A—H5A	119.3	H10D—C10A—H10E	109.5
C2B—C3B—C4B	120.7 (4)	H10D—C10A—H10F	109.5
C2B—C3B—H3B	119.6	H10E—C10A—H10F	109.5
C4B—C3B—H3B	119.6	C26A—C25A—H25A	107.8

C6B—C5B—H5B	119.4	C24A—C25A—H25A	107.8
C4B—C5B—C6B	121.3 (3)	C24A—C25A—C26A	108.3 (4)
C4B—C5B—H5B	119.4	C29A—C25A—H25A	107.8
N2A—C9A—H9A	119.0	C29A—C25A—C26A	116.1 (5)
N2A—C9A—C8A	122.0 (4)	C29A—C25A—C24A	108.7 (6)
C8A—C9A—H9A	119.0	C22A—C23A—H23C	110.2
C3A—C2A—C1A	119.7 (4)	C22A—C23A—H23D	110.2
C3A—C2A—H2A	120.1	H23C—C23A—H23D	108.5
C1A—C2A—H2A	120.1	C24A—C23A—C22A	107.6 (4)
C8A—C21A—H21A	109.9	C24A—C23A—H23C	110.2
C8A—C21A—C22A	107.8 (3)	C24A—C23A—H23D	110.2
C8A—C21A—C19A	110.4 (3)	C26B—C25B—H25B	107.3
C22A—C21A—H21A	109.9	C29B—C25B—C26B	115.4 (4)
C19A—C21A—H21A	109.9	C29B—C25B—H25B	107.3
C19A—C21A—C22A	108.8 (3)	C29B—C25B—C24B	112.2 (5)
C7A—C8A—C9A	120.3 (4)	C24B—C25B—C26B	106.8 (4)
C7A—C8A—C21A	122.1 (4)	C24B—C25B—H25B	107.3
C9A—C8A—C21A	117.5 (4)	C13B—C14B—H14B	119.8
C12A—C13A—H13A	119.6	C13B—C14B—C15B	120.4 (5)
C14A—C13A—C12A	120.7 (4)	C15B—C14B—H14B	119.8
C14A—C13A—H13A	119.6	C33B—C34B—H34B	119.6
O1A—C4A—C3A	114.6 (4)	C35B—C34B—C33B	120.8 (5)
O1A—C4A—C5A	125.3 (4)	C35B—C34B—H34B	119.6
C5A—C4A—C3A	120.1 (4)	N1A—C26A—C25A	110.4 (5)
C12A—C11A—C7A	125.0 (4)	N1A—C26A—H26C	109.6
C12A—C11A—C20A	117.3 (4)	N1A—C26A—H26D	109.6
C20A—C11A—C7A	117.6 (4)	C25A—C26A—H26C	109.6
C12B—C11B—C7B	124.2 (4)	C25A—C26A—H26D	109.6
C12B—C11B—C20B	119.1 (4)	H26C—C26A—H26D	108.1
C20B—C11B—C7B	116.6 (4)	C20B—C15B—C16B	115.9 (7)
C11B—C12B—H12B	119.8	C14B—C15B—C20B	119.5 (6)
C11B—C12B—C13B	120.3 (5)	C14B—C15B—C16B	124.5 (6)
C13B—C12B—H12B	119.8	C25A—C24A—C23A	109.7 (4)
N2B—C9B—C8B	122.8 (4)	C25A—C24A—H24A	110.8
N2B—C9B—H9B	118.6	C23A—C24A—H24A	110.8
C8B—C9B—H9B	118.6	C28A—C24A—C25A	107.5 (5)
N1A—C22A—C21A	111.4 (3)	C28A—C24A—C23A	107.2 (5)
N1A—C22A—H22A	107.2	C28A—C24A—H24A	110.8
N1A—C22A—C23A	110.2 (3)	C35A—C34A—C33A	120.9 (4)
C21A—C22A—H22A	107.2	C35A—C34A—H34A	119.6
C23A—C22A—C21A	113.4 (4)	C33A—C34A—H34A	119.6
C23A—C22A—H22A	107.2	N1B—C27B—H27A	109.4
C19B—C20B—C11B	121.6 (4)	N1B—C27B—H27B	109.4
C19B—C20B—C15B	119.7 (5)	N1B—C27B—C28B	111.0 (4)
C15B—C20B—C11B	118.7 (6)	H27A—C27B—H27B	108.0
C20A—C19A—C21A	117.0 (4)	C28B—C27B—H27A	109.4

C18A—C19A—C21A	122.7 (4)	C28B—C27B—H27B	109.4
C18A—C19A—C20A	119.5 (4)	C29B—C30B—H30A	124 (3)
C13A—C14A—H14A	120.0	C29B—C30B—H30B	123 (4)
C13A—C14A—C15A	120.1 (4)	H30A—C30B—H30B	112 (5)
C15A—C14A—H14A	120.0	C22B—C23B—H23A	110.3
N2B—C31B—H31A	109.6	C22B—C23B—H23B	110.3
N2B—C31B—H31B	109.6	H23A—C23B—H23B	108.6
N2B—C31B—C32B	110.3 (3)	C24B—C23B—C22B	106.9 (4)
H31A—C31B—H31B	108.1	C24B—C23B—H23A	110.3
C32B—C31B—H31A	109.6	C24B—C23B—H23B	110.3
C32B—C31B—H31B	109.6	C25B—C24B—C23B	109.4 (4)
N1B—C22B—H22B	107.0	C25B—C24B—H24B	110.2
N1B—C22B—C21B	111.6 (4)	C25B—C24B—C28B	108.8 (5)
N1B—C22B—C23B	110.6 (4)	C23B—C24B—H24B	110.2
C21B—C22B—H22B	107.0	C28B—C24B—C23B	108.0 (5)
C23B—C22B—H22B	107.0	C28B—C24B—H24B	110.2
C23B—C22B—C21B	113.2 (4)	C19B—C18B—H18B	119.6
C8B—C21B—C22B	107.7 (3)	C19B—C18B—C17B	120.8 (8)
C8B—C21B—H21B	109.2	C17B—C18B—H18B	119.6
C22B—C21B—H21B	109.2	C25A—C29A—H29A	113.2
C19B—C21B—C8B	110.7 (4)	C30A—C29A—C25A	133.7 (9)
C19B—C21B—C22B	110.9 (4)	C30A—C29A—H29A	113.2
C19B—C21B—H21B	109.2	N1A—C27A—H27C	109.2
C19A—C20A—C11A	120.3 (4)	N1A—C27A—H27D	109.2
C15A—C20A—C11A	119.9 (4)	N1A—C27A—C28A	112.1 (4)
C15A—C20A—C19A	119.8 (4)	H27C—C27A—H27D	107.9
N2A—C31A—H31C	109.4	C28A—C27A—H27C	109.2
N2A—C31A—H31D	109.4	C28A—C27A—H27D	109.2
N2A—C31A—C32A	111.1 (3)	C15B—C16B—H16B	119.0
H31C—C31A—H31D	108.0	C17B—C16B—C15B	121.9 (6)
C32A—C31A—H31C	109.4	C17B—C16B—H16B	119.0
C32A—C31A—H31D	109.4	C24A—C28A—C27A	107.6 (4)
C14A—C15A—C16A	122.2 (4)	C24A—C28A—H28C	110.2
C20A—C15A—C14A	119.2 (4)	C24A—C28A—H28D	110.2
C20A—C15A—C16A	118.6 (4)	C27A—C28A—H28C	110.2
C20B—C19B—C21B	117.6 (4)	C27A—C28A—H28D	110.2
C18B—C19B—C20B	120.5 (6)	H28C—C28A—H28D	108.5
C18B—C19B—C21B	121.5 (6)	C27B—C28B—H28A	110.3
C37B—C32B—C31B	119.8 (4)	C27B—C28B—H28B	110.3
C37B—C32B—C33B	120.1 (4)	C24B—C28B—C27B	107.2 (4)
C33B—C32B—C31B	120.1 (4)	C24B—C28B—H28A	110.3
C19A—C18A—H18A	119.8	C24B—C28B—H28B	110.3
C19A—C18A—C17A	120.4 (5)	H28A—C28B—H28B	108.5
C17A—C18A—H18A	119.8	C18B—C17B—H17B	119.8
N1B—C26B—H26A	109.4	C16B—C17B—C18B	120.4 (6)
N1B—C26B—H26B	109.4	C16B—C17B—H17B	119.8

N1B—C26B—C25B	111.3 (4)	C29A—C30A—H30C	120.0
H26A—C26B—H26B	108.0	C29A—C30A—H30D	120.0
C25B—C26B—H26A	109.4	H30C—C30A—H30D	120.0
O1B—C4B—C3B—C2B	176.8 (3)	C12B—C13B—C14B—C15B	-6.4 (7)
O1B—C4B—C5B—C6B	-179.8 (3)	C9B—N2B—C1B—C2B	173.7 (3)
N2B—C31B—C32B—C37B	133.0 (4)	C9B—N2B—C1B—C6B	-5.0 (5)
N2B—C31B—C32B—C33B	-48.3 (5)	C9B—N2B—C31B—C32B	97.7 (4)
N2A—C1A—C2A—C3A	-176.1 (3)	C9B—C8B—C21B—C22B	-83.5 (5)
N2A—C9A—C8A—C7A	1.5 (6)	C9B—C8B—C21B—C19B	155.1 (4)
N2A—C9A—C8A—C21A	177.8 (3)	C22A—N1A—C26A—C25A	-51.6 (5)
N2A—C31A—C32A—C37A	130.3 (4)	C22A—N1A—C27A—C28A	66.6 (5)
N2A—C31A—C32A—C33A	-49.4 (6)	C22A—C21A—C8A—C7A	88.3 (4)
N1B—C22B—C21B—C8B	57.1 (5)	C22A—C21A—C8A—C9A	-87.9 (4)
N1B—C22B—C21B—C19B	178.4 (4)	C22A—C21A—C19A—C20A	-78.0 (4)
N1B—C22B—C23B—C24B	-20.2 (6)	C22A—C21A—C19A—C18A	91.6 (5)
N1B—C26B—C25B—C29B	109.7 (4)	C22A—C23A—C24A—C25A	-44.1 (5)
N1B—C26B—C25B—C24B	-15.9 (5)	C22A—C23A—C24A—C28A	72.3 (6)
N1B—C27B—C28B—C24B	-13.7 (7)	C20B—C11B—C12B—C13B	8.7 (6)
C6B—C7B—C8B—C9B	-9.9 (5)	C20B—C19B—C18B—C17B	1.1 (8)
C6B—C7B—C8B—C21B	175.1 (3)	C20B—C15B—C16B—C17B	-2.0 (9)
C6B—C7B—C11B—C12B	25.1 (6)	C19A—C21A—C8A—C7A	-30.4 (5)
C6B—C7B—C11B—C20B	-150.5 (4)	C19A—C21A—C8A—C9A	153.3 (3)
C7B—C6B—C1B—N2B	-4.1 (5)	C19A—C21A—C22A—N1A	-178.9 (3)
C7B—C6B—C1B—C2B	177.3 (3)	C19A—C21A—C22A—C23A	-53.9 (4)
C7B—C6B—C5B—C4B	-178.4 (3)	C19A—C20A—C15A—C14A	-169.9 (4)
C7B—C8B—C9B—N2B	0.9 (6)	C19A—C20A—C15A—C16A	8.1 (6)
C7B—C8B—C21B—C22B	91.6 (5)	C19A—C18A—C17A—C16A	4.7 (9)
C7B—C8B—C21B—C19B	-29.8 (5)	C14A—C15A—C16A—C17A	176.8 (5)
C7B—C11B—C12B—C13B	-166.8 (4)	C31B—N2B—C1B—C2B	-12.8 (5)
C7B—C11B—C20B—C19B	-16.8 (6)	C31B—N2B—C1B—C6B	168.5 (3)
C7B—C11B—C20B—C15B	162.4 (4)	C31B—N2B—C9B—C8B	-167.0 (3)
N1A—C22A—C23A—C24A	-23.0 (5)	C31B—C32B—C37B—C36B	178.8 (4)
N1A—C27A—C28A—C24A	-16.5 (7)	C31B—C32B—C33B—C34B	-179.0 (4)
C1B—N2B—C9B—C8B	6.7 (5)	C22B—N1B—C26B—C25B	-50.7 (5)
C1B—N2B—C31B—C32B	-75.9 (4)	C22B—N1B—C27B—C28B	66.1 (6)
C1B—C2B—C3B—C4B	1.2 (5)	C22B—C21B—C19B—C20B	-82.1 (5)
C1B—C6B—C7B—C8B	11.3 (5)	C22B—C21B—C19B—C18B	90.7 (5)
C1B—C6B—C7B—C11B	-173.3 (3)	C22B—C23B—C24B—C25B	-47.3 (6)
C1B—C6B—C5B—C4B	4.7 (5)	C22B—C23B—C24B—C28B	70.9 (6)
C12A—C13A—C14A—C15A	-7.1 (6)	C21B—C8B—C9B—N2B	176.2 (3)
C12A—C11A—C20A—C19A	167.1 (3)	C21B—C22B—C23B—C24B	-146.3 (4)
C12A—C11A—C20A—C15A	-13.1 (5)	C21B—C19B—C18B—C17B	-171.6 (5)
C7A—C6A—C1A—N2A	-4.2 (5)	C20A—C19A—C18A—C17A	2.4 (7)
C7A—C6A—C1A—C2A	177.7 (3)	C20A—C15A—C16A—C17A	-1.1 (7)
C7A—C6A—C5A—C4A	-178.4 (3)	C31A—N2A—C1A—C6A	173.8 (3)

C7A—C11A—C20A—C19A	-15.1 (5)	C31A—N2A—C1A—C2A	-8.1 (5)
C7A—C11A—C20A—C15A	164.7 (3)	C31A—N2A—C9A—C8A	-172.4 (3)
C8B—C7B—C11B—C12B	-159.6 (4)	C31A—C32A—C33A—C34A	179.9 (5)
C8B—C7B—C11B—C20B	24.9 (5)	C19B—C20B—C15B—C14B	-172.3 (4)
C8B—C21B—C19B—C20B	37.4 (5)	C19B—C20B—C15B—C16B	8.0 (6)
C8B—C21B—C19B—C18B	-149.8 (5)	C19B—C18B—C17B—C16B	5.2 (10)
C6A—C7A—C8A—C9A	-9.2 (5)	C32B—C37B—C36B—C35B	0.0 (7)
C6A—C7A—C8A—C21A	174.6 (3)	C32B—C33B—C34B—C35B	0.6 (7)
C6A—C7A—C11A—C12A	27.5 (6)	C18A—C19A—C20A—C11A	171.0 (4)
C6A—C7A—C11A—C20A	-150.1 (4)	C18A—C19A—C20A—C15A	-8.8 (6)
C6A—C1A—C2A—C3A	2.1 (5)	C18A—C17A—C16A—C15A	-5.4 (9)
C6A—C5A—C4A—O1A	-178.7 (3)	C26B—N1B—C22B—C21B	-160.8 (4)
C6A—C5A—C4A—C3A	-0.7 (5)	C26B—N1B—C22B—C23B	72.2 (5)
C1A—N2A—C9A—C8A	5.4 (5)	C26B—N1B—C27B—C28B	-51.8 (6)
C1A—N2A—C31A—C32A	-80.1 (4)	C26B—C25B—C24B—C23B	67.1 (5)
C1A—C6A—C5A—C4A	3.6 (5)	C26B—C25B—C24B—C28B	-50.7 (5)
C5A—C6A—C1A—N2A	173.9 (3)	C10B—O1B—C4B—C3B	178.6 (3)
C5A—C6A—C1A—C2A	-4.2 (5)	C10B—O1B—C4B—C5B	-2.4 (6)
C3B—C2B—C1B—N2B	-175.8 (3)	C37A—C32A—C33A—C34A	0.2 (7)
C3B—C2B—C1B—C6B	2.8 (5)	C37A—C36A—C35A—C34A	-0.4 (8)
C3B—C4B—C5B—C6B	-0.9 (5)	C32A—C37A—C36A—C35A	1.3 (7)
C5B—C6B—C7B—C8B	-165.6 (3)	C32A—C33A—C34A—C35A	0.7 (8)
C5B—C6B—C7B—C11B	9.8 (5)	C36A—C37A—C32A—C31A	179.1 (4)
C5B—C6B—C1B—N2B	172.9 (3)	C36A—C37A—C32A—C33A	-1.2 (7)
C5B—C6B—C1B—C2B	-5.7 (5)	C36A—C35A—C34A—C33A	-0.6 (9)
C5B—C4B—C3B—C2B	-2.2 (6)	C37B—C32B—C33B—C34B	-0.3 (6)
C9A—N2A—C1A—C6A	-3.8 (5)	C37B—C36B—C35B—C34B	0.3 (7)
C9A—N2A—C1A—C2A	174.3 (3)	C36B—C35B—C34B—C33B	-0.6 (7)
C9A—N2A—C31A—C32A	97.6 (4)	C13B—C14B—C15B—C20B	1.3 (7)
C2A—C3A—C4A—O1A	176.6 (3)	C13B—C14B—C15B—C16B	-179.0 (5)
C2A—C3A—C4A—C5A	-1.6 (6)	C29B—C25B—C24B—C23B	-60.3 (5)
C21A—C22A—C23A—C24A	-148.7 (4)	C29B—C25B—C24B—C28B	-178.1 (4)
C21A—C19A—C20A—C11A	-19.1 (5)	C33B—C32B—C37B—C36B	0.0 (6)
C21A—C19A—C20A—C15A	161.1 (4)	C10A—O1A—C4A—C3A	-179.6 (3)
C21A—C19A—C18A—C17A	-166.9 (5)	C10A—O1A—C4A—C5A	-1.5 (6)
C8A—C7A—C6A—C1A	10.5 (5)	C25A—C24A—C28A—C27A	68.4 (6)
C8A—C7A—C6A—C5A	-167.5 (3)	C23A—C24A—C28A—C27A	-49.4 (7)
C8A—C7A—C11A—C12A	-156.8 (4)	C25B—C24B—C28B—C27B	67.4 (6)
C8A—C7A—C11A—C20A	25.6 (5)	C14B—C15B—C16B—C17B	178.4 (6)
C8A—C21A—C22A—N1A	61.3 (4)	C26A—N1A—C22A—C21A	-158.7 (4)
C8A—C21A—C22A—C23A	-173.7 (3)	C26A—N1A—C22A—C23A	74.5 (5)
C8A—C21A—C19A—C20A	40.1 (5)	C26A—N1A—C27A—C28A	-49.4 (6)
C8A—C21A—C19A—C18A	-150.3 (4)	C26A—C25A—C24A—C23A	65.0 (5)
C13A—C12A—C11A—C7A	-171.7 (3)	C26A—C25A—C24A—C28A	-51.2 (5)
C13A—C12A—C11A—C20A	5.9 (5)	C26A—C25A—C29A—C30A	19.2 (11)
C13A—C14A—C15A—C20A	-0.2 (6)	C15B—C20B—C19B—C21B	165.1 (4)

C13A—C14A—C15A—C16A	-178.0 (4)	C15B—C20B—C19B—C18B	-7.8 (7)
C4A—C3A—C2A—C1A	0.9 (6)	C15B—C16B—C17B—C18B	-4.6 (11)
C11A—C12A—C13A—C14A	4.1 (6)	C24A—C25A—C26A—N1A	-14.6 (5)
C11A—C7A—C6A—C1A	-173.7 (3)	C24A—C25A—C29A—C30A	141.6 (9)
C11A—C7A—C6A—C5A	8.3 (5)	C27B—N1B—C22B—C21B	81.2 (4)
C11A—C7A—C8A—C9A	174.7 (3)	C27B—N1B—C22B—C23B	-45.8 (5)
C11A—C7A—C8A—C21A	-1.5 (5)	C27B—N1B—C26B—C25B	70.0 (5)
C11A—C20A—C15A—C14A	10.3 (6)	C30B—C29B—C25B—C26B	17.7 (8)
C11A—C20A—C15A—C16A	-171.7 (4)	C30B—C29B—C25B—C24B	140.4 (6)
C11B—C7B—C8B—C9B	174.4 (3)	C23B—C22B—C21B—C8B	-177.4 (4)
C11B—C7B—C8B—C21B	-0.6 (5)	C23B—C22B—C21B—C19B	-56.1 (6)
C11B—C12B—C13B—C14B	1.3 (6)	C23B—C24B—C28B—C27B	-51.2 (7)
C11B—C20B—C19B—C21B	-15.7 (6)	C29A—C25A—C26A—N1A	108.0 (6)
C11B—C20B—C19B—C18B	171.4 (4)	C29A—C25A—C24A—C23A	-62.0 (5)
C11B—C20B—C15B—C14B	8.5 (6)	C29A—C25A—C24A—C28A	-178.2 (4)
C11B—C20B—C15B—C16B	-171.2 (4)	C27A—N1A—C22A—C21A	84.2 (4)
C12B—C11B—C20B—C19B	167.4 (4)	C27A—N1A—C22A—C23A	-42.6 (5)
C12B—C11B—C20B—C15B	-13.4 (6)	C27A—N1A—C26A—C25A	66.8 (5)
